# Noise effect on soliton phenomena in fractional stochastic Kraenkel-Manna-Merle system arising in ferromagnetic materials

**DOI:** 10.1038/s41598-024-52211-3

**Published:** 2024-01-20

**Authors:** Humaira Yasmin, Azzh Saad Alshehry, Abdul Hamid Ganie, Ahmad Shafee, Rasool Shah

**Affiliations:** 1 Department of Basic Sciences, General Administration of Preparatory Year, King Faisal University, Al-Ahsa, 31982 Saudi Arabia; 2 Department of Mathematical Sciences, Faculty of Sciences, Princess Nourah Bint Abdulrahman University, P.O. Box 84428, Riyadh, 11671 Saudi Arabia; 3 Basic Science Department, College of Science and Theoretical Studies, Saudi Electronic University, Riyadh, 11673 Saudi Arabia; 4 PAAET, College of Technological Studies, Laboratory Technology Department, Shuwaikh, 70654 Kuwait; 5 Department of Mathematics, Abdul Wali Khan University, Mardan, Pakistan

**Keywords:** Engineering, Mathematics and computing

## Abstract

This work dives into the Conformable Stochastic Kraenkel-Manna-Merle System (CSKMMS), an important mathematical model for exploring phenomena in ferromagnetic materials. A wide spectrum of stochastic soliton solutions that include hyperbolic, trigonometric and rational functions, is generated using a modified version of Extended Direct Algebraic Method (EDAM) namely *r*+mEDAM. These stochastic soliton solutions have practical relevance for describing magnetic field behaviour in zero-conductivity ferromagnets. By using Maple to generate 2D and 3D graphical representations, the study analyses how stochastic terms and noise impact these soliton solutions. Finally, this study adds to our knowledge of magnetic field behaviour in ferromagnetic materials by shedding light on the effect of noise on soliton processes inside the CSKMMS.

## Introduction

Stochastic Fractional Partial Differential Equations (SFPDEs), a subcategory of Fractional Partial Differential Equations (FPDEs), are a solid mathematical foundation with numerous applications in science and engineering^1–3^. These equations combine stochastic processes, which handle unpredictability, with fractional calculus, which addresses memory and non-local effects. This unusual mix enables them to mimic sophisticated occurrences that are beyond the scope of traditional differential equations. SFDEs are useful in finance for tasks like asset price modelling and risk assessment. Furthermore, they serve an important role in subjects such as physics, biology, geophysics, and environmental research, improving understanding of a wide range of natural processes. Furthermore, SFPDEs provide significant contributions to control theory, signal processing, and image analysis by proposing novel solutions to complicated issues in system and data analysis.

Mathematicians have been heavily involved in researching Partial Differential Equations (PDEs) and FPDEs to investigate numerical, analytical, travelling, and soliton solutions^4–9^. Among these investigations, solitons in the setting of Nonlinear FPDEs (NFPDEs) have long captivated physicists and mathematics professionals alike. Numerous analytical techniques, such as the the Sardar sub-equation method^10^, tan-function method^11^, the (G’/G)-expansion approach^12^, the Khater method^13^, the sub-equation method^14^, the Kudryashov method^15^, Jacobi elliptic function method^16^, Bilinear method^17^, mEDAM^18^ method and the exp-function method^19^ have been developed to elucidate and characterise soliton behaviours within NFPDEs. mEDAM^20,21^ has emerged as a promising strategy that can be used to both NPDEs and NFPDEs. This approach uses a transformational mechanism to convert NFPDEs or NPDEs into Nonlinear Ordinary Differential Equations (NODEs), which are then solved with series-based solutions. The resultant NODE is then used to construct a set of algebraic equations that, when solved, offer soliton solutions to the FPDE at hand. mEDAM is notable for its extraordinary ability to develop a larger range of soliton solution families.

In this study, we take into account the CSKMMS which is articulated as^22^:

1 $$\begin{aligned} &D^{\alpha }_x u_t-u D^{\alpha }_x z+\kappa D^{\alpha }_x u=\varrho B_t(t) D^{\alpha }_x u,\\&D^{\alpha }_x z_t-u D^{\alpha }_x u=\varrho B_t(t) D^{\alpha }_x z,\\ \end{aligned}$$

where $u \equiv u(x,t)$ denotes magnetization, $z \equiv z(x,t)$ denotes outer magnetic fields and are related to the ferrite, $\kappa$ represents damping coefficient,$\varrho$ is the noise intensity, *B*(*t*) is the Brownian motion and the operator $D^\alpha _x(\cdot )$ denote conformables partial derivatives. Nguepjouo et al.^23^ investigated the application of magnetization density expansion and coordinate transformations to convert the structure to the following:

2 $$\begin{aligned} &u_{xt}-u z_x+\kappa u_x=0,\\&z_{xt}-u u_x =0.\\ \end{aligned}$$

This description encompasses the nonlinear propagation of short waves in saturated ferromagnetic materials with zero conductivity. We get the Kraenkel-Manna-Merle system (KMMS) in (2) from the CSKMMS in (1) by setting $\varrho = 0$ and $\alpha =1$. When damping is removed ($\kappa =0$), Equation (2) becomes integrable and displays Lax pairings. Numerous scholars have devised various approaches over the years to generate solutions for both the CSKMMS (1) and KMMS (2), taking into account varying values of $\kappa$. Among these approaches are the bilinear method^23^, inverse scattering method^24^, (G’/G)-expansion method^25^, new auxiliary equation method^26^, auxiliary equation method^27^, and F-expansion technique^28^. The goal of this study is to use the *r*+mEDAM technique to construct stochastic soliton solutions for the CSKMMS given in (1), with the special constraint of $\kappa = 0$. These solutions are set to provide physicists with significant insights into fundamental physical events. Furthermore, we use the Maple programme to produce a plethora of graphical representations, allowing for a more in-depth investigation of the effect of noise on the stochastic soliton solutions inside the CSKMMS. Recent publications across various domains encapsulate a diverse spectrum of scientific investigations. Zhang et al.^29^ explore a Ferrotoroidic candidate characterized by distinct spin chains, while Huang et al.^30^ focus on fault diagnosis of bearings in wind turbine gearboxes under real operating conditions with limited noisy data labels. Wang et al.^31^ propose a universal estimation method for Tire-Pavement Interaction Noise (TPIN) using 3D image technology, contributing to sustainability efforts. In nonlinear dynamics, Li and Kai^32^ delve into wave structures and chaotic behaviors of the cubic-quartic nonlinear Schrodinger equation in birefringent fibers. Additionally, Li et al.^33^ introduce a Dilatancy Equation based on property-dependent plastic potential theory for geomaterials in the context of fractals and fractions. Furthermore, Hu et al.^34^ discuss consensus control of general linear multiagent systems with antagonistic interactions and communication noises, while Wang et al.^35^ evaluate road traffic noise exposure considering differential crowd characteristics in transportation research. These diverse studies underscore the multidisciplinary nature and broad scope of recent scientific endeavors.

The other part of this study is structured as follows: Section "Methodology and resources" presents Brownian motion, conformable fractal derivative definitions, and the approach of *r*+mEDAM. Section "Wave equation for CSKMMS" focuses on deriving the wave equation for CSKMMS, while Section "Stochastic soliton solutions For CSKMMS" uses the *r*+mEDAM to derive stochastic soliton solutions for CSKMMS. Section "Discussion and graphs" presents a series of graphs illustrating the influence of multiplicative noise on CSKMMS’s soliton solutions. Finally, in the final section, we synthesise our findings and give conclusions.

## Methodology and resources

### Brownian motion

The stochastic process ${W(t)}_{t\ge 0}$ is called a Brownian motion if it satisfies the following conditions^36^:

- $$B(0)=0$$,
- *B*(*t*) is continuous function,
- $B(t)-B(y)$ is independent for $s < t$,
- $B(t)-B(y)$ has a Gaussian distribution with variance $t - y$ and mean 0.

### Conformable derivative

Researchers have established various dissentions for fractional derivatives^37–41^. However, We can address FPDEs by leveraging the supremacy of conformable derivative. The stochastic soliton solutions of CFKMMS given in (1), for instance, cannot be obtained using alternative fractional derivative formulations because they do not obey the chain rule^28,42^. As a result, in equation (1), the derivatives utilised are corresponded to conformable derivatives. The operator that represents these derivatives of order $\alpha$ is defined in^43^ as follows:

3 $$\begin{aligned} D^\alpha _\phi z(\phi )= \lim _{\omega \rightarrow 0} \frac{z(\omega \phi ^{1-\alpha }+\phi )-z(\phi )}{\omega }, \quad \alpha \in (0,1]. \end{aligned}$$

The following features of this derivative are used in this investigation:

4 $$\begin{aligned}{} & {} D^\alpha _\phi \phi ^\gamma = \gamma \phi ^{\gamma -\alpha }, \end{aligned}$$

5 $$\begin{aligned}{} & {} D^\alpha _\phi (\gamma _1\eta (\phi )\pm \gamma _2\theta (\phi ))=\gamma _1D^\alpha _\phi (\eta (\phi ))\pm \gamma _2D^\alpha _\phi (\theta (\phi )), \end{aligned}$$

6 $$\begin{aligned}{} & {} D^\alpha _\phi \chi [\zeta (\phi )] = \chi '_\zeta (\zeta (\phi ))D^\alpha _\phi \zeta (\phi ), \end{aligned}$$

where $\eta (\phi )$, $\theta (\phi )$, $\chi (\phi )$ & $\zeta (\phi )$ represent functions that exhibit differentiability, whereas $\gamma$, $\gamma _1$ & $\gamma _2$ signify constants.

### The operational mechanism of *r*+mEDAM

This section introduces and expands on the *r*+mEDAM technique. We start with a nonlinear FPDE in the style shown below^18,20,21^:

7 $$\begin{aligned} E(D^\beta _t u, D^\alpha _x u, u, u D^\alpha _x u, \ldots )=0, \end{aligned}$$

where $u=u(x,t)$. To solve (7), the subsequently listed procedures are used:

1. First, a variable transformation of the form $u(x,t)=U(\phi )$, where $\phi$ can be defined in different ways, is performed. Equation (7) is transformed into the following NODE by the application of this transformation:
  8 $$\begin{aligned} F(U', UU', U \dots )=0, \end{aligned}$$
  The primes in (8) represent derivatives of *U* with respect to $\phi$. Equation (8) may require one or more integration stages to get the constants of integration.
2. As a result, we propose that (8) has the following solution:
  9 $$\begin{aligned} U(\phi )=\sum _{i=0}^{N}b_i(r+\Lambda (\phi ))^i. \end{aligned}$$
  The parameters $b_i$ (where $i = 0, \ldots , N$) are to be driven later, and $\Lambda (\phi )$ meets the following NODE:
  10 $$\begin{aligned} \Lambda '(\phi )=\ln (\tau )(j+k \Lambda (\phi )+l(\Lambda (\phi ))^2), \end{aligned}$$
  where $\tau \ne 0,1$ & *j*, *k*, *l* are constants.
3. We may calculate the positive integer *N* by seeking a homogeneous balance between the major nonlinear component and the highest order derivative in Equation (8).
4. Our next step is to plug (9) into (8), or the equation obtained by integrating (8), with the goal of building a polynomial expression in $\Lambda (\phi )$. Following that, we organise all expressions involving $\Lambda (\phi )$ consistently. This allows us to set the coefficients of the resultant polynomial to zero, resulting in a system of nonlinear algebraic equations involving $b_i$ (where $i = 0, \ldots , N$) and other important factors.
5. We use the MAPLE programme to solve this system of algebraic equations.
6. After evaluating the unknown parameters and inserting them together with the solutions for $\Lambda (\phi )$ into equation (9), the soliton solutions for (7) are constructed. We may infer families of soliton solutions using the general solution supplied by equation (10), as shown below:

#### Family 1

For $$G<0 \quad l\ne 0$$,

$$\begin{aligned} \Lambda _1(\phi )= & {} -{\frac{k}{2l}}+{\frac{\sqrt{-G}\tan _\tau \left( \frac{1}{2}\sqrt{-G }\phi \right) }{2l}}\\ \Lambda _2(\phi )= & {} -{\frac{k}{2l}}-{\frac{\sqrt{-G}\cot _\tau \left( \frac{1}{2}\sqrt{-G }\phi \right) }{2l}}\\ \Lambda _3(\phi )= & {} -{\frac{k}{2l}}+{\frac{\sqrt{-G} \left( \tan _\tau \left( \sqrt{-G}\phi \right) \pm \left( \sec _\tau \left( \sqrt{-G}\phi \right) \right) \right) }{2l}}\\ \Lambda _4(\phi )= & {} -{\frac{k}{2l}}-{\frac{\sqrt{-G} \left( \cot _\tau \left( \sqrt{-G}\phi \right) \pm \left( \csc _\tau \left( \sqrt{-G}\phi \right) \right) \right) }{2l}} \end{aligned}$$

&

$$\begin{aligned} \Lambda _5(\phi )=-{\frac{k}{2l}}+{\frac{\sqrt{-G} \left( \tan _\tau \left( \frac{1}{4} \sqrt{-G}\phi \right) -\cot _\tau \left( \frac{1}{4}\sqrt{-G}\phi \right) \right) }{4c}} \end{aligned}$$

#### Family 2

For $$G>0 \quad l\ne 0$$,

$$\begin{aligned} \Lambda _6(\phi )= & {} -{\frac{k}{2l}}-{\frac{\sqrt{G}\tanh _\tau \left( \frac{1}{2}\sqrt{G} \phi \right) }{2l}}\\ \Lambda _7(\phi )= & {} -{\frac{k}{2l}}-{\frac{\sqrt{G}\coth _\tau \left( \frac{1}{2}\sqrt{G} \phi \right) }{2l}}\\ \Lambda _8(\phi )= & {} -{\frac{k}{2l}}-{\frac{\sqrt{G} \left( \tanh _\tau \left( \sqrt{G}\phi \right) \pm \left( { sech_\tau } \left( \sqrt{G}\phi \right) \right) \right) }{2l}}\\ \Lambda _9(\phi )= & {} -{\frac{k}{2l}}-{\frac{\sqrt{G} \left( \coth _\tau \left( \sqrt{G}\phi \right) \pm \left( { csch_\tau } \left( \sqrt{G}\phi \right) \right) \right) }{2l}} \end{aligned}$$

&

$$\begin{aligned} \Lambda _{10}(\phi )=-{\frac{k}{2l}}-{\frac{\sqrt{G} \left( \tanh _\tau \left( \frac{1}{4} \sqrt{G}\phi \right) -\coth _\tau \left( \frac{1}{4}\sqrt{G}\phi \right) \right) }{4l}} \end{aligned}$$

#### Family 3

For $$jl>0$$ & $$k=0$$,

$$\begin{aligned} \Lambda _{11}(\phi )= & {} \sqrt{{\frac{j}{l}}}\tan _\tau \left( \sqrt{jl}\phi \right) \\ \Lambda _{12}(\phi )= & {} -\sqrt{{\frac{j}{l}}}\cot _\tau \left( \sqrt{jl}\phi \right) \\ \Lambda _{13}(\phi )= & {} \sqrt{{\frac{j}{l}}} \left( \tan _\tau \left( 2\,\sqrt{jl}\phi \right) \pm \left( \sec _\tau \left( 2\,\sqrt{jl}\phi \right) \right) \right) \\ \Lambda _{14}(\phi )= & {} -\sqrt{{\frac{j}{l}}} \left( \cot _\tau \left( 2\,\sqrt{jl}\phi \right) \pm \left( \csc _\tau \left( 2\,\sqrt{jl}\phi \right) \right) \right) \end{aligned}$$

&

$$\begin{aligned} \Lambda _{15}(\phi )=\frac{1}{2}\sqrt{{\frac{j}{l}}} \left( \tan _\tau \left( \frac{1}{2}\sqrt{jl}\phi \right) -\cot _\tau \left( \frac{1}{2}\sqrt{jl}\phi \right) \right) \end{aligned}$$

#### Family 4

For $$jl<0$$ & $$k=0$$,

$$\begin{aligned} \Lambda _{16}(\phi )= & {} -\sqrt{-{\frac{j}{l}}}\tanh _\tau \left( \sqrt{-jl}\phi \right) \\ \Lambda _{17}(\phi )= & {} -\sqrt{-{\frac{j}{l}}}\coth _\tau \left( \sqrt{-jl}\phi \right) \\ \Lambda _{18}(\phi )= & {} -\sqrt{-{\frac{j}{l}}} \left( \tanh _\tau \left( 2\,\sqrt{-jl}\phi \right) \pm \left( i{ sech_\tau } \left( 2\, \sqrt{-jl}\phi \right) \right) \right) \\ \Lambda _{19}(\phi )= & {} -\sqrt{-{\frac{j}{l}}} \left( \coth _\tau \left( 2\,\sqrt{-jl}\phi \right) \pm \left( { csch_\tau } \left( 2\, \sqrt{-jl}\phi \right) \right) \right) \end{aligned}$$

&

$$\begin{aligned} \Lambda _{20}(\phi )=-\frac{1}{2}\sqrt{-{\frac{j}{l}}} \left( \tanh _\tau \left( \frac{1}{2}\sqrt{-jl}\phi \right) +\coth _\tau \left( \frac{1}{2}\sqrt{-jl}\phi \right) \right) \end{aligned}$$

#### Family 5

For $$l=j$$ & $$k=0$$,

$$\begin{aligned} \Lambda _{21}(\phi )= & {} \tan _\tau \left( j\phi \right) \\ \Lambda _{22}(\phi )= & {} -\cot _\tau \left( j\phi \right) \\ \Lambda _{23}(\phi )= & {} \tan _\tau \left( 2\,j\phi \right) \pm \left( \sec _\tau \left( 2\,j\phi \right) \right) \\ \Lambda _{24}(\phi )= & {} -\cot _\tau \left( 2\,j\phi \right) \pm \left( \csc _\tau \left( 2\,j\phi \right) \right) \end{aligned}$$

&

$$\begin{aligned} \Lambda _{25}(\phi )=\frac{1}{2}\tan _\tau \left( \frac{1}{2}j\phi \right) -\frac{1}{2}\cot _\tau \left( \frac{1}{2}j\phi \right) \end{aligned}$$

#### Family 6

For $$l=-j$$ & $$k=0$$,

$$\begin{aligned} \Lambda _{26}(\phi )= & {} -\tanh _\tau \left( j\phi \right) \\ \Lambda _{27}(\phi )= & {} -\coth _\tau \left( j\phi \right) \\ \Lambda _{28}(\phi )= & {} -\tanh _\tau \left( 2\,j\phi \right) \pm \left( i { sech_\tau } \left( 2\,j\phi \right) \right) \\ \Lambda _{29}(\phi )= & {} -\coth _\tau \left( 2\,j\phi \right) \pm \left( { csch_\tau } \left( 2\,j\phi \right) \right) \end{aligned}$$

&

$$\begin{aligned} \Lambda _{30}(\phi )=-\frac{1}{2}\tanh _\tau \left( \frac{1}{2}j\phi \right) -\frac{1}{2}\coth _\tau \left( \frac{1}{2}j\phi \right) \end{aligned}$$

#### Family 7

For $$G=0$$,

$$\begin{aligned} \Lambda _{31}(\phi )=-2\,{\frac{j \left( k\phi \,{\ln \tau }+2 \right) }{{k}^{2}\phi \,{\ln \tau } }} \end{aligned}$$

#### Family 8

For $$k=\eta$$, $$j=n\eta (n\ne 0)$$ & $$l=0$$,:

$$\begin{aligned} \Lambda _{32}(\phi )={\tau }^{\eta ,\phi }-n \end{aligned}$$

#### Family 9

For $$k=l=0$$,

$$\begin{aligned} \Lambda _{33}(\phi )=j\phi \,{\ln (\tau )} \end{aligned}$$

#### Family 10

For $$k=j=0$$,

$$\begin{aligned} \Lambda _{34}(\phi )=-{\frac{1}{l\phi \,{\ln (\tau )}}} \end{aligned}$$

#### Family 11

For $$j=0$$, $$k\ne 0$$ & $$l\ne 0$$,

$$\begin{aligned} \Lambda _{35}(\phi )=-{\frac{k}{l \left( \cosh _\tau \left( k\phi \right) -\sinh _\tau \left( k\phi \right) +1 \right) }} \end{aligned}$$

&

$$\begin{aligned} \Lambda _{36}(\phi )=-{\frac{k \left( \cosh _\tau \left( k\phi \right) +\sinh _\tau \left( k\phi \right) \right) }{l \left( \cosh _\tau \left( k\phi \right) +\sinh _\tau \left( k \phi \right) +1 \right) }} \end{aligned}$$

#### Family 12

For $$k=\eta$$, $$l=n\eta (n\ne 0)$$ & $$j=0$$,

$$\begin{aligned} \Lambda _{37}(\phi )={\frac{{\tau }^{\phi \eta }}{1-n{\tau }^{\phi \eta }}}. \end{aligned}$$

Here $G = k^2 - 4jl$. Let us now look at the generalisation of hyperbolic and trigonometric functions which are defined as:

$$\begin{aligned} &\sin _\tau \left( \phi \right) ={\frac{{\Lambda }^{I\phi }-{\Lambda }^{-I\phi }}{2I}}, \quad \cos _\tau \left( \phi \right) =\frac{{\Lambda }^{I\phi }+{\Lambda }^{-I\phi }}{2},\\&\sec _\tau \left( \phi \right) =\frac{1}{\cos _\tau \left( \phi \right) }, \quad \csc _\tau \left( \phi \right) =\frac{1}{\sin _\tau \left( \phi \right) },\\&\tan _\tau \left( \phi \right) =\frac{\sin _\tau \left( \phi \right) }{\cos _\tau \left( \phi \right) }, \quad \cot _\tau \left( \phi \right) =\frac{\cos _\tau \left( \phi \right) }{\sin _\tau \left( \phi \right) },\\ \end{aligned}$$

Similarly,

$$\begin{gathered} \sinh _{\tau } \left( \phi \right) = \frac{{\Lambda ^{\phi } - \Lambda ^{{ - \phi }} }}{2},\quad \hfill \\ \cosh _{\tau } \left( \phi \right) = \frac{{\Lambda ^{\phi } + \Lambda ^{{ - \phi }} }}{2}, \hfill \\ \text{sech} _{\tau } \left( \phi \right) = \frac{1}{{\cosh _{\tau } \left( \phi \right)}},\quad \hfill \\ csch_{\tau } \left( \phi \right) = \frac{1}{{\sinh _{\tau } \left( \phi \right)}}, \hfill \\ \tanh _{\tau } \left( \phi \right) = \frac{{\sinh _{\tau } \left( \phi \right)}}{{\cosh _{\tau } \left( \phi \right)}}, \hfill \\ \coth _{\tau } \left( \phi \right) = \frac{{\cosh _{\tau } \left( \phi \right)}}{{\sinh _{\tau } \left( \phi \right)}}. \hfill \\ \end{gathered}$$

## Wave equation for CSKMMS

To get the wave equation of CSKMMS (1), we consider the damping effect $\kappa =0$ and utilize the following wave transformation:

11 $$\begin{aligned}&u(x,t)=U(\phi ) e^{\left(\varrho B(t)-\frac{\varrho ^2t}{2}\right)},\\&z(x,t)=Z(\phi ) e^{\left(\varrho B(t)-\frac{\varrho ^2t}{2}\right)}, \\&\phi = \frac{\lambda x^\alpha }{\alpha }+\sigma t,\\ \end{aligned}$$

where $U(\phi )$ and $Z(\phi )$ are real functions, $\lambda$ and $\sigma$ are nonzero constants, and we are able to obtain the wave equation of the CSKMMS (1). We note that

12 $$\begin{aligned}&D^\alpha _x u=\lambda U{^\prime} e^{\left(\varrho B(t)-\frac{\varrho ^2t}{2}\right)}, \quad D^\alpha _x (u_t)=[\lambda \sigma U^{\prime\prime}+\varrho \lambda U{^\prime} B_t] e^{\left(\varrho B(t)-\frac{\varrho ^2t}{2}\right)},\\&D^\alpha _x z=\lambda Z{^\prime} e^{\left(\varrho B(t)-\frac{\varrho ^2t}{2}\right)}, \quad D^\alpha _x (u_t)=[\lambda \sigma Z^{\prime\prime}+\varrho \lambda Z{^\prime} B_t] e^{\left(\varrho B(t)-\frac{\varrho ^2t}{2}\right)}, \\ \end{aligned}$$

where $\frac{\varrho ^2t}{2}$ is the Itô correction term. Inserting (11) into (1) and using (12), we have

13 $$\begin{aligned}&\lambda \sigma U^{\prime\prime}-\lambda U Z{^\prime} e^{\left(\varrho B(t)-\frac{\varrho ^2t}{2}\right)}=0, \\&\lambda \sigma Z^{\prime\prime}-\lambda UU{^\prime} e^{\left(\varrho B(t)-\frac{\varrho ^2t}{2}\right)}=0. \\ \end{aligned}$$

Taking expectation on both sides of the equations in (13), we have

14 $$\begin{aligned}&\sigma U^{\prime\prime}- U Z{^\prime} \mathbb {E}( e^{(\varrho B(t))}) e^{\left(-\frac{\varrho ^2t}{2}\right)}=0, \\&\sigma Z^{\prime\prime}-UU{^\prime} \mathbb {E}( e^{(\varrho B(t))}) e^{\left(-\frac{\varrho ^2t}{2}\right)}=0. \\ \end{aligned}$$

We note that $\mathbb {E}(e^{\varrho B(t)}=e^{\frac{\varrho ^2t}{2}})$, where *B*(*t*) is normal standard distribution and $\varrho$ is a real constant. Now, (14) has the form

15 $$\begin{aligned}&\sigma U^{\prime\prime}- U Z{^\prime}=0, \\&\sigma Z^{\prime\prime}- UU{^\prime}=0. \\ \end{aligned}$$

We get the following result after performing single integration on the second equation in (15):

16 $$\begin{aligned} Z'=\frac{U^2}{2\sigma }+\frac{C}{\sigma }, \end{aligned}$$

where *C* represents an integration constant. By substituting (16) into the first equation in (15), we get:

17 $$\begin{aligned} U''-\frac{1}{2\sigma ^2}U^3-\frac{C}{\sigma ^2}U=0 \end{aligned}$$

## Stochastic soliton solutions For CSKMMS

By generating a condition of homogeneous balance between $U''$ and $U^3$ present in (17), we deduce that *N* = 1. By inserting $N=1$ into the equation (9) we arrive at the following series-based solutions for (17):

18 $$\begin{aligned} U(\xi )=\sum _{i=0}^{1}b_i(r+\Lambda (\phi ))^i=b_0+b_1(r+\Lambda (\phi ))^1. \end{aligned}$$

By putting (18) into (17) and stacking terms with equivalent powers of $\Lambda (\phi )$, we have an expression in $\Lambda (\phi )$. When the coefficients are set to zero, the process generates a system of nonlinear algebraic equations. When Maple is used to solve this system, the two sets of solutions offered are as follows:

### Case 1

19 $$\begin{aligned} \begin{aligned} b_{{0}}=-\sigma \,\ln \bigg ( \tau \bigg ) \bigg ( -2\,rl+k \bigg ),b _{{1}}=-2\,\ln \bigg ( \tau \bigg ) l\sigma ,r=r,\sigma =\sigma ,C=-\frac{1}{2} \, \bigg ( \ln \bigg ( \tau \bigg ) {\sigma } \bigg ) ^{2}G. \end{aligned} \end{aligned}$$

### Case 2

20 $$\begin{aligned} \begin{aligned} b_{{0}}= \bigg ( -2\,\sqrt{2}lr+\sqrt{2}k \bigg ) \sqrt{-{\frac{C}{G}}},b_{{1}}=2\,\sqrt{-{\frac{2C}{G}}}l,r=r,\sigma =\frac{1}{\ln ( \tau )}\sqrt{-{\frac{2C}{G}}}, C=C. \end{aligned} \end{aligned}$$

Considering case. 1 into (19), and utilizing (11), (16) and (18) together with the corresponding general solution of (10), we get the following families of stochastic soliton solutions for (1):

#### Family 1.1

When $$G<0 \quad l\ne 0$$,

21 $$\begin{aligned}{} & {} \begin{aligned} u_{1,1}(x,t)&= e^{\left(\varrho B(t)-\frac{\varrho ^2 t}{2}\right)} \bigg ( -\sigma \,\ln \bigg ( \tau \bigg ) \bigg ( -2\,rl+k \bigg )\\&\quad -2\,\ln \bigg ( \tau \bigg ) l\sigma \, \bigg ( r-\frac{1}{2} \,{\frac{k}{l}}+\frac{1}{2} \,{ \frac{\sqrt{-G}\tan \bigg ( \frac{1}{2} \,\sqrt{-G}\phi \bigg ) }{l}} \bigg ) \bigg ),\\ z_{1,1}(x,t)&=e^{\left(\varrho B(t)-\frac{\varrho ^2 t}{2}\right)} \bigg ( -\frac{1}{2} \,{\frac{ \bigg ( \ln \bigg ( \tau \bigg ) \bigg ) ^{2}G\tan \bigg ( \frac{1}{2} \,\sqrt{-G}\phi \bigg ) }{\sqrt{-G}}}\\&\quad +\frac{1}{2} \,{\frac{ \bigg ( \ln \bigg ( \tau \bigg ) \bigg ) ^{2}G\arctan \bigg ( \tan \bigg ( \frac{1}{2} \,\sqrt{-G}\phi \bigg ) \bigg ) }{\sqrt{-G}}}+{\frac{C \phi }{\sigma }} \bigg ),\\ \end{aligned} \end{aligned}$$

22 $$\begin{aligned}{} & {} \begin{aligned} u_{1,2}(x,t)&=e^{\left(\varrho B(t)-\frac{\varrho ^2 t}{2}\right)} \bigg ( -\sigma \,\ln \bigg ( \tau \bigg ) \bigg ( -2\,rl+k \bigg ) -2\,\ln \bigg ( \tau \bigg ) l\sigma \, \bigg ( r-\frac{1}{2} \,{\frac{k}{l}}-\frac{1}{2} \,{ \frac{\sqrt{-G}\cot \bigg ( \frac{1}{2} \,\sqrt{-G}\phi \bigg ) }{l}} \bigg ) \bigg ),\\ z_{1,2}(x,t)&=e^{\left(\varrho B(t)-\frac{\varrho ^2 t}{2}\right)} \bigg ( \frac{1}{2} \,{\frac{ \bigg ( \ln \bigg ( \tau \bigg ) \bigg ) ^{2}G\cot \bigg ( \frac{1}{2} \,\sqrt{-G}\phi \bigg ) }{\sqrt{-G}}}\\&\quad -\frac{1}{4} \,{\frac{ \bigg ( \ln \bigg ( \tau \bigg ) \bigg ) ^{2}G\pi }{\sqrt{-G}}}+\frac{1}{2} \,{\frac{ \bigg ( \ln \bigg ( \tau \bigg ) \bigg ) ^{2}G{ arccot} \bigg ( \cot \bigg ( \frac{1}{2} \,\sqrt{-G}\phi \bigg ) \bigg ) }{\sqrt{-G}} }+{\frac{C\phi }{\sigma }} \bigg ),\\ \end{aligned} \end{aligned}$$

23 $$\begin{aligned}{} & {} \begin{aligned} u_{1,3}(x,t)&=e^{\left(\varrho B(t)-\frac{\varrho ^2 t}{2}\right)} \bigg ( -\sigma \,\ln \bigg ( \tau \bigg ) \bigg ( -2\,rl+k \bigg ) \\&\quad -2\,\ln \bigg ( \tau \bigg ) l\sigma \, \bigg ( r-\frac{1}{2} \,{\frac{k}{l}}+\frac{1}{2} \,{ \frac{\sqrt{-G} \bigg ( \tan \bigg ( \sqrt{-G}\phi \bigg ) +\sec \bigg ( \sqrt{-G}\phi \bigg ) \bigg ) }{l}} \bigg ) \bigg ),\\ z_{1,3}(x,t)&=e^{\left(\varrho B(t)-\frac{\varrho ^2 t}{2}\right)} \bigg ( -\frac{1}{4} \,{\frac{ \bigg ( \ln \bigg ( \tau \bigg ) \bigg ) ^{2}G\tan \bigg ( \sqrt{-G}\phi \bigg ) }{\sqrt{-G}}}+\frac{1}{4} \, \bigg ( \ln \bigg ( \tau \bigg ) \bigg ) ^{2}G\phi \\&\quad -\frac{1}{2} \,{\frac{ \bigg ( \ln \bigg ( \tau \bigg ) \bigg ) ^{2}G}{\sqrt{-G}\cos \bigg ( \sqrt{-G}\phi \bigg ) }}-\frac{1}{4} \,{\frac{ \bigg ( \ln \bigg ( \tau \bigg ) \bigg ) ^{2}G\sin \bigg ( \sqrt{-G}\phi \bigg ) }{\sqrt{- G}\cos \bigg ( \sqrt{-G}\phi \bigg ) }}+{\frac{C\phi }{\sigma }} \bigg ),\\ \end{aligned} \end{aligned}$$

24 $$\begin{aligned}{} & {} \begin{aligned} u_{1,4}(x,t)&=e^{\left(\varrho B(t)-\frac{\varrho ^2 t}{2}\right)} \bigg ( -\sigma \,\ln \bigg ( \tau \bigg ) \bigg ( -2\,rl+k \bigg ) \\&\quad -2\,\ln \bigg ( \tau \bigg ) l\sigma \, \bigg ( r-\frac{1}{2} \,{\frac{k}{l}}-\frac{1}{2} \,{ \frac{\sqrt{-G} \bigg ( \cot \bigg ( \sqrt{-G}\phi \bigg ) +\csc \bigg ( \sqrt{-G}\phi \bigg ) \bigg ) }{l}} \bigg ) \bigg ),\\ z_{1,4}(x,t)&=e^{\left(\varrho B(t)-\frac{\varrho ^2 t}{2}\right)} \bigg ( \frac{1}{4} \,{\frac{ \bigg ( \ln \bigg ( \tau \bigg ) \bigg ) ^{2}G\cot \bigg ( \sqrt{-G}\phi \bigg ) }{\sqrt{-G}}}+\frac{1}{4} \, \bigg ( \ln \bigg ( \tau \bigg ) \bigg ) ^{2}G\phi \\&\quad +\frac{1}{2} \,{\frac{ \bigg ( \ln \bigg ( \tau \bigg ) \bigg ) ^{2}G}{\sqrt{-G}\sin \bigg ( \sqrt{-G}\phi \bigg ) }}\\&\quad +\frac{1}{4} \,{\frac{ \bigg ( \ln \bigg ( \tau \bigg ) \bigg ) ^{2}G\cos \bigg ( \sqrt{-G}\phi \bigg ) }{\sqrt{- G}\sin \bigg ( \sqrt{-G}\phi \bigg ) }}+{\frac{C\phi }{\sigma }} \bigg ),\\ \end{aligned} \end{aligned}$$

and

25 $$\begin{aligned} \begin{aligned} u_{1,5}(x,t)&=e^{\left(\varrho B(t)-\frac{\varrho ^2 t}{2}\right)} \bigg ( -\sigma \,\ln \bigg ( \tau \bigg ) \bigg ( -2\,rl+k \bigg ) \\&\quad -2\,\ln \bigg ( \tau \bigg ) l\sigma \, \bigg ( r-\frac{1}{2} \,{\frac{k}{l}}+\frac{1}{4} \,{ \frac{\sqrt{-G} \bigg ( \tan \bigg ( \frac{1}{4} \,\sqrt{-G}\phi \bigg ) -\cot \bigg ( \frac{1}{4} \,\sqrt{-G}\phi \bigg ) \bigg ) }{l}} \bigg ) \bigg ),\\ z_{1,5}(x,t)&=e^{\left(\varrho B(t)-\frac{\varrho ^2 t}{2}\right)} \bigg ( -\frac{1}{4} \,{\frac{ \bigg ( \ln \bigg ( \tau \bigg ) \bigg ) ^{2}G\tan \bigg ( \frac{1}{4} \,\sqrt{-G}\phi \bigg ) }{\sqrt{-G}}}+\frac{1}{4} \, \bigg ( \ln \bigg ( \tau \bigg ) \bigg ) ^{2}G\phi \\&\quad +\frac{1}{4} \,{\frac{ \bigg ( \ln \bigg ( \tau \bigg ) \bigg ) ^{2}G\cot \bigg ( \frac{1}{4} \,\sqrt{-G}\phi \bigg ) }{\sqrt{-G}}}+{\frac{C\phi }{\sigma }} \bigg ).\\ \end{aligned} \end{aligned}$$

#### Family 1.2

When $$G>0 \quad l\ne 0$$,

26 $$\begin{aligned}{} & {} \begin{aligned} u_{1,6}(x,t)&=e^{\left(\varrho B(t)-\frac{\varrho ^2 t}{2}\right)} \bigg ( -\sigma \,\ln \bigg ( \tau \bigg ) \bigg ( -2\,rl+k \bigg ) \\&\quad -2\,\ln \bigg ( \tau \bigg ) l\sigma \, \bigg ( r-\frac{1}{2} \,{\frac{k}{l}}-\frac{1}{2} \,{ \frac{\sqrt{G}\tanh \bigg ( \frac{1}{2} \,\sqrt{G}\phi \bigg ) }{l}} \bigg ) \bigg ),\\ z_{1,6}(x,t)&=e^{\left(\varrho B(t)-\frac{\varrho ^2 t}{2}\right)} \bigg ( -\frac{1}{2} \,\sqrt{G} \bigg ( \ln \bigg ( \tau \bigg ) \bigg ) ^{2}\tanh \bigg ( \frac{1}{2} \,\sqrt{G}\phi \bigg ) \\&\quad -\frac{1}{4} \,\sqrt{G} \bigg ( \ln \bigg ( \tau \bigg ) \bigg ) ^{2}\ln \bigg ( \tanh \bigg ( \frac{1}{2} \,\sqrt{G}\phi \bigg ) -1 \bigg )\\&\quad +\frac{1}{4} \,\sqrt{G} \bigg ( \ln \bigg ( \tau \bigg ) \bigg ) ^{2}\ln \bigg ( \tanh \bigg ( \frac{1}{2} \,\sqrt{G}\phi \bigg ) +1 \bigg ) +{\frac{C\phi }{\sigma }} \bigg ),\\ \end{aligned} \end{aligned}$$

27 $$\begin{aligned}{} & {} \begin{aligned} u_{1,7}(x,t)&=e^{\left(\varrho B(t)-\frac{\varrho ^2 t}{2}\right)} \bigg ( -\sigma \,\ln \bigg ( \tau \bigg ) \bigg ( -2\,rl+k \bigg ) \\&\quad -2\,\ln \bigg ( \tau \bigg ) l\sigma \, \bigg ( r-\frac{1}{2} \,{\frac{k}{l}}-\frac{1}{2} \,{ \frac{\sqrt{G}\coth \bigg ( \frac{1}{2} \,\sqrt{G}\phi \bigg ) }{l}} \bigg ) \bigg ),\\ z_{1,7}(x,t)&=e^{\left(\varrho B(t)-\frac{\varrho ^2 t}{2}\right)} \bigg ( -\frac{1}{2} \,\sqrt{G} \bigg ( \ln \bigg ( \tau \bigg ) \bigg ) ^{2}\coth \bigg ( \frac{1}{2} \,\sqrt{G}\phi \bigg ) \\&\quad -\frac{1}{4} \,\sqrt{G} \bigg ( \ln \bigg ( \tau \bigg ) \bigg ) ^{2}\ln \bigg ( \coth \bigg ( \frac{1}{2} \,\sqrt{G}\phi \bigg ) -1 \bigg )\\&\quad +\frac{1}{4} \,\sqrt{G} \bigg ( \ln \bigg ( \tau \bigg ) \bigg ) ^{2}\ln \bigg ( \coth \bigg ( \frac{1}{2} \,\sqrt{G}\phi \bigg ) +1 \bigg ) +{\frac{C\phi }{\sigma }} \bigg ),\\ \end{aligned} \end{aligned}$$

28 $$\begin{aligned}{} & {} \begin{aligned} u_{1,8}(x,t)&=e^{\left(\varrho B(t)-\frac{\varrho ^2 t}{2}\right)} \bigg ( -\sigma \,\ln \bigg ( \tau \bigg ) \bigg ( -2\,rl+k \bigg )\\&\quad -2\,\ln \bigg ( \tau \bigg ) l\sigma \, \bigg ( r-\frac{1}{2} \,{\frac{k}{l}}-\frac{1}{2} \,{ \frac{\sqrt{G} \bigg ( \tanh \bigg ( \sqrt{G}\phi \bigg ) +{ I} \,{ sech} \bigg ( \sqrt{G}\phi \bigg ) \bigg ) }{l}} \bigg ) \bigg ),\\ z_{1,8}(x,t)&=e^{\left(\varrho B(t)-\frac{\varrho ^2 t}{2}\right)} \bigg ( \frac{1}{4} \, \bigg ( \ln \bigg ( \tau \bigg ) \bigg ) ^{2}G\phi \\&\quad -\frac{1}{4} \,\sqrt{G } \bigg ( \ln \bigg ( \tau \bigg ) \bigg ) ^{2}\tanh \bigg ( \sqrt{G} \phi \bigg ) +\frac{1}{2} \,{\frac{\sqrt{G} \bigg ( \ln \bigg ( \tau \bigg ) \bigg ) ^{2}{} { I}\, \bigg ( \sinh \bigg ( \sqrt{G}\phi \bigg ) \bigg ) ^{2}}{\cosh \bigg ( \sqrt{G}\phi \bigg ) }}\\&\quad -\frac{1}{2} \, \sqrt{G} \bigg ( \ln \bigg ( \tau \bigg ) \bigg ) ^{2}{} { I}\, \cosh \bigg ( \sqrt{G}\phi \bigg ) +\frac{1}{4} \,{\frac{\sqrt{G} \bigg ( \ln \bigg ( \tau \bigg ) \bigg ) ^{2}{{ I}}^{2}\sinh \bigg ( \sqrt{G}\phi \bigg ) }{\cosh \bigg ( \sqrt{G}\phi \bigg ) }}+ {\frac{C\phi }{\sigma }} \bigg ),\\ \end{aligned} \end{aligned}$$

29 $$\begin{aligned}{} & {} \begin{aligned} u_{1,9}(x,t)&=e^{\left(\varrho B(t)-\frac{\varrho ^2 t}{2}\right)} \bigg ( -\sigma \,\ln \bigg ( \tau \bigg ) \bigg ( -2\,rl+k \bigg ) \\&\quad -2\,\ln \bigg ( \tau \bigg ) l\sigma \, \bigg ( r-\frac{1}{2} \,{\frac{k}{l}}-\frac{1}{2} \,{ \frac{\sqrt{G} \bigg ( \coth \bigg ( \sqrt{G}\phi \bigg ) + { csch} \bigg ( \sqrt{G}\phi \bigg ) \bigg ) }{l}} \bigg ) \bigg ),\\ z_{1,9}(x,t)&=e^{\left(\varrho B(t)-\frac{\varrho ^2 t}{2}\right)} \bigg ( \frac{1}{4} \, \bigg ( \ln \bigg ( \tau \bigg ) \bigg ) ^{2}G\phi \\&\quad -\frac{1}{4} \,\sqrt{G } \bigg ( \ln \bigg ( \tau \bigg ) \bigg ) ^{2}\coth \bigg ( \sqrt{G} \phi \bigg ) -\frac{1}{2} \,{\frac{\sqrt{G} \bigg ( \ln \bigg ( \tau \bigg ) \bigg ) ^{2} \bigg ( \cosh \bigg ( \sqrt{G}\phi \bigg ) \bigg ) ^{2}}{\sinh \bigg ( \sqrt{G}\phi \bigg ) }}\\&\quad +\frac{1}{2} \,\sqrt{G} \bigg ( \ln \bigg ( \tau \bigg ) \bigg ) ^{2}\sinh \bigg ( \sqrt{G}\phi \bigg ) -\frac{1}{4} \,{\frac{\sqrt{G} \bigg ( \ln \bigg ( \tau \bigg ) \bigg ) ^{2}\cosh \bigg ( \sqrt{G}\phi \bigg ) }{\sinh \bigg ( \sqrt{G}\phi \bigg ) }}+{\frac{C\phi }{\sigma }} \bigg ),\\ \end{aligned} \end{aligned}$$

and

30 $$\begin{aligned} \begin{aligned} u_{1,10}(x,t)&=e^{\left(\varrho B(t)-\frac{\varrho ^2 t}{2}\right)} \bigg ( -\sigma \,\ln \bigg ( \tau \bigg ) \bigg ( -2\,rl+k \bigg )\\&\quad -2\,\ln \bigg ( \tau \bigg ) l\sigma \, \bigg ( r-\frac{1}{2} \,{\frac{k}{l}}-\frac{1}{4} \,{ \frac{\sqrt{G} \bigg ( \tanh \bigg ( \frac{1}{4} \,\sqrt{G}\phi \bigg ) -\coth \bigg ( \frac{1}{4} \,\sqrt{G}\phi \bigg ) \bigg ) }{l}} \bigg ) \bigg ),\\ z_{1,10}(x,t)&=e^{\left(\varrho B(t)-\frac{\varrho ^2 t}{2}\right)} \bigg ( -\frac{1}{4} \,\sqrt{G} \bigg ( \ln \bigg ( \tau \bigg ) \bigg ) ^{2}\tanh \bigg ( \frac{1}{4} \,\sqrt{G}\phi \bigg )\\&\quad -\frac{1}{4} \,\sqrt{G} \bigg ( \ln \bigg ( \tau \bigg ) \bigg ) ^{2}\coth \bigg ( \frac{1}{4} \,\sqrt{G}\phi \bigg ) +{ \frac{C\phi }{\sigma }} \bigg ).\\ \end{aligned} \end{aligned}$$

#### Family 1.3

When $$lj>0$$ and $$k=0$$,

31 $$\begin{aligned}{} & {} \begin{aligned} u_{1,11}(x,t)&=e^{\left(\varrho B(t)-\frac{\varrho ^2 t}{2}\right)} \bigg ( 2\,\sigma \,\ln \bigg ( \tau \bigg ) rl-2\,\ln \bigg ( \tau \bigg ) l \sigma \, \bigg ( r+\sqrt{{\frac{j}{l}}}\tan \bigg ( \sqrt{jl}\phi \bigg ) \bigg ) \bigg ),\\ z_{1,11}(x,t)&=e^{\left(\varrho B(t)-\frac{\varrho ^2 t}{2}\right)} \bigg ( {\frac{ \bigg ( \ln \bigg ( \tau \bigg ) \bigg ) ^{2}lj\tan \bigg ( \sqrt{jl}\phi \bigg ) }{\sqrt{jl}}}\\&\quad -{\frac{ \bigg ( \ln \bigg ( \tau \bigg ) \bigg ) ^{2}\arctan \bigg ( \tan \bigg ( \sqrt{jl}\phi \bigg ) \bigg ) jl}{\sqrt{jl}}}+{\frac{C\phi }{\sigma }} \bigg ),\\ \end{aligned} \end{aligned}$$

32 $$\begin{aligned}{} & {} \begin{aligned} u_{1,12}(x,t)&=e^{\left(\varrho B(t)-\frac{\varrho ^2 t}{2}\right)} \bigg ( 2\,\sigma \,\ln \bigg ( \tau \bigg ) rl-2\,\ln \bigg ( \tau \bigg ) l \sigma \, \bigg ( r-\sqrt{{\frac{j}{l}}}\cot \bigg ( \sqrt{jl}\phi \bigg ) \bigg ) \bigg ),\\ z_{1,12}(x,t)&=e^{\left(\varrho B(t)-\frac{\varrho ^2 t}{2}\right)} \bigg ( -{\frac{ \bigg ( \ln \bigg ( \tau \bigg ) \bigg ) ^{2}lj\cot \bigg ( \sqrt{jl}\phi \bigg ) }{\sqrt{jl}}}\\&\quad +\frac{1}{2} \,{\frac{ \bigg ( \ln \bigg ( \tau \bigg ) \bigg ) ^{2}lj\pi }{\sqrt{jl}}}-{\frac{ \bigg ( \ln \bigg ( \tau \bigg ) \bigg ) ^{2}lj{ arccot} \bigg ( \cot \bigg ( \sqrt{jl}\phi \bigg ) \bigg ) }{\sqrt{jl}}}+{\frac{C \phi }{\sigma }} \bigg ),\\ \end{aligned} \end{aligned}$$

33 $$\begin{aligned}{} & {} \begin{aligned} u_{1,13}(x,t)&=e^{\left(\varrho B(t)-\frac{\varrho ^2 t}{2}\right)} \bigg ( 2\,\sigma \,\ln \bigg ( \tau \bigg ) rl-2\,\ln \bigg ( \tau \bigg ) l \sigma \, \bigg ( r+\sqrt{{\frac{j}{l}}} \bigg ( \tan \bigg ( 2\,\sqrt{ jl}\phi \bigg ) +\sec \bigg ( 2\,\sqrt{jl}\phi \bigg ) \bigg ) \bigg ) \bigg ),\\ z_{1,13}(x,t)&=e^{\left(\varrho B(t)-\frac{\varrho ^2 t}{2}\right)} \bigg ( \frac{1}{2} \,{\frac{ \bigg ( \ln \bigg ( \tau \bigg ) \bigg ) ^{2}lj\tan \bigg ( 2\,\sqrt{jl}\phi \bigg ) }{\sqrt{jl}}}- \bigg ( \ln \bigg ( \tau \bigg ) \bigg ) ^{2}lj\phi \\&\quad +{\frac{ \bigg ( \ln \bigg ( \tau \bigg ) \bigg ) ^{2}lj}{\sqrt{jl}\cos \bigg ( 2\,\sqrt{jl }\phi \bigg ) }}+\frac{1}{2} \,{\frac{ \bigg ( \ln \bigg ( \tau \bigg ) \bigg ) ^{2}lj\sin \bigg ( 2\,\sqrt{jl}\phi \bigg ) }{\sqrt{jl} \cos \bigg ( 2\,\sqrt{jl}\phi \bigg ) }}+{\frac{C\phi }{\sigma }} \bigg ),\\ \end{aligned} \end{aligned}$$

34 $$\begin{aligned}{} & {} \begin{aligned} u_{1,14}(x,t)&=e^{\left(\varrho B(t)-\frac{\varrho ^2 t}{2}\right)} \bigg ( 2\,\sigma \,\ln \bigg ( \tau \bigg ) rl-2\,\ln \bigg ( \tau \bigg ) l \sigma \, \bigg ( r-\sqrt{{\frac{j}{l}}} \bigg ( \cot \bigg ( 2\,\sqrt{ jl}\phi \bigg ) +\csc \bigg ( 2\,\sqrt{jl}\phi \bigg ) \bigg ) \bigg ) \bigg ),\\ z_{1,14}(x,t)&=e^{\left(\varrho B(t)-\frac{\varrho ^2 t}{2}\right)} \bigg ( -\frac{1}{2} \,{\frac{ \bigg ( \ln \bigg ( \tau \bigg ) \bigg ) ^{2}lj\cot \bigg ( 2\,\sqrt{jl}\phi \bigg ) }{\sqrt{jl}}}- \bigg ( \ln \bigg ( \tau \bigg ) \bigg ) ^{2}lj\phi \\&\quad -{\frac{ \bigg ( \ln \bigg ( \tau \bigg ) \bigg ) ^{2}lj}{\sqrt{jl}\sin \bigg ( 2\,\sqrt{jl }\phi \bigg ) }}-\frac{1}{2} \,{\frac{ \bigg ( \ln \bigg ( \tau \bigg ) \bigg ) ^{2}lj\cos \bigg ( 2\,\sqrt{jl}\phi \bigg ) }{\sqrt{jl} \sin \bigg ( 2\,\sqrt{jl}\phi \bigg ) }}+{\frac{C\phi }{\sigma }} \bigg ),\\ \end{aligned} \end{aligned}$$

and

35 $$\begin{aligned} \begin{aligned} u_{1,15}(x,t)&=e^{\left(\varrho B(t)-\frac{\varrho ^2 t}{2}\right)} \bigg ( 2\,\sigma \,\ln \bigg ( \tau \bigg ) rl-2\,\ln \bigg ( \tau \bigg ) l \sigma \, \bigg ( r+\frac{1}{2} \,\sqrt{{\frac{j}{l}}} \bigg ( \tan \bigg ( \frac{1}{2} \, \sqrt{jl}\phi \bigg ) -\cot \bigg ( \frac{1}{2} \,\sqrt{jl}\phi \bigg ) \bigg ) \bigg ) \bigg ),\\ z_{1,15}(x,t)&=e^{\left(\varrho B(t)-\frac{\varrho ^2 t}{2}\right)} \bigg ( \frac{1}{2} \,{\frac{ \bigg ( \ln \bigg ( \tau \bigg ) \bigg ) ^{2}lj\tan \bigg ( \frac{1}{2} \,\sqrt{jl}\phi \bigg ) }{\sqrt{jl}}}\\&\quad - \bigg ( \ln \bigg ( \tau \bigg ) \bigg ) ^{2}lj\phi -\frac{1}{2} \,{\frac{ \bigg ( \ln \bigg ( \tau \bigg ) \bigg ) ^{2}lj\cot \bigg ( \frac{1}{2} \,\sqrt{jl}\phi \bigg ) }{\sqrt{jl}}}+{\frac{C\phi }{\sigma }} \bigg ).\\ \end{aligned} \end{aligned}$$

#### Family 1.4

When $$lj>0$$ and $$k=0$$,

36 $$\begin{aligned}{} & {} \begin{aligned} u_{1,16}(x,t)&=e^{\left(\varrho B(t)-\frac{\varrho ^2 t}{2}\right)} \bigg ( 2\,\sigma \,\ln \bigg ( \tau \bigg ) rl-2\,\ln \bigg ( \tau \bigg ) l \sigma \, \bigg ( r-\sqrt{-{\frac{j}{l}}}\tanh \bigg ( \sqrt{-jl}\phi \bigg ) \bigg ) \bigg ),\\ z_{1,16}(x,t)&=e^{\left(\varrho B(t)-\frac{\varrho ^2 t}{2}\right)} \bigg ( {\frac{ \bigg ( \ln \bigg ( \tau \bigg ) \bigg ) ^{2}lj\tanh \bigg ( \sqrt{-jl}\phi \bigg ) }{\sqrt{-jl}}}+\frac{1}{2} \,{\frac{ \bigg ( \ln \bigg ( \tau \bigg ) \bigg ) ^{2}\ln \bigg ( \tanh \bigg ( \sqrt{-jl} \phi \bigg ) -1 \bigg ) jl}{\sqrt{-jl}}}\\&\quad -\frac{1}{2} \,{\frac{ \bigg ( \ln \bigg ( \tau \bigg ) \bigg ) ^{2}\ln \bigg ( \tanh \bigg ( \sqrt{-jl} \phi \bigg ) +1 \bigg ) jl}{\sqrt{-jl}}}+{\frac{C\phi }{\sigma }} \bigg ),\\ \end{aligned} \end{aligned}$$

37 $$\begin{aligned}{} & {} \begin{aligned} u_{1,17}(x,t)&=e^{\left(\varrho B(t)-\frac{\varrho ^2 t}{2}\right)} \bigg ( 2\,\sigma \,\ln \bigg ( \tau \bigg ) rl-2\,\ln \bigg ( \tau \bigg ) l \sigma \, \bigg ( r-\sqrt{-{\frac{j}{l}}}\coth \bigg ( \sqrt{-jl}\phi \bigg ) \bigg ) \bigg ),\\ z_{1,17}(x,t)&=e^{\left(\varrho B(t)-\frac{\varrho ^2 t}{2}\right)} \bigg ( {\frac{ \bigg ( \ln \bigg ( \tau \bigg ) \bigg ) ^{2}lj\coth \bigg ( \sqrt{-jl}\phi \bigg ) }{\sqrt{-jl}}}+\frac{1}{2} \,{\frac{ \bigg ( \ln \bigg ( \tau \bigg ) \bigg ) ^{2}\ln \bigg ( \coth \bigg ( \sqrt{-jl} \phi \bigg ) -1 \bigg ) jl}{\sqrt{-jl}}}\\&\quad -\frac{1}{2} \,{\frac{ \bigg ( \ln \bigg ( \tau \bigg ) \bigg ) ^{2}\ln \bigg ( \coth \bigg ( \sqrt{-jl} \phi \bigg ) +1 \bigg ) jl}{\sqrt{-jl}}}+{\frac{C\phi }{\sigma }} \bigg ),\\ \end{aligned} \end{aligned}$$

38 $$\begin{aligned}{} & {} \begin{aligned} u_{1,18}(x,t)&=e^{\left(\varrho B(t)-\frac{\varrho ^2 t}{2}\right)} \bigg ( 2\,\sigma \,\ln \bigg ( \tau \bigg ) rl-2\,\ln \bigg ( \tau \bigg ) l \sigma \, \bigg ( r-\sqrt{-{\frac{j}{l}}} \\&\quad \bigg ( \tanh \bigg ( 2\, \sqrt{-jl}\phi \bigg ) +{ I}\,{ sech} \bigg ( 2\, \sqrt{-jl}\phi \bigg ) \bigg ) \bigg ) \bigg ),\\ z_{1,18}(x,t)&=e^{\left(\varrho B(t)-\frac{\varrho ^2 t}{2}\right)} \bigg ( - \bigg ( \ln \bigg ( \tau \bigg ) \bigg ) ^{2}lj\phi +\frac{1}{2} \,{\frac{ \bigg ( \ln \bigg ( \tau \bigg ) \bigg ) ^{2}lj\tanh \bigg ( 2\,\sqrt{-jl}\phi \bigg ) }{\sqrt{-jl}}}\\&\quad -{\frac{ \bigg ( \ln \bigg ( \tau \bigg ) \bigg ) ^{2}lj{ I}\, \bigg ( \sinh \bigg ( 2\, \sqrt{-jl}\phi \bigg ) \bigg ) ^{2}}{\sqrt{-jl}\cosh \bigg ( 2\, \sqrt{-jl}\phi \bigg ) }}\\&\quad +{\frac{ \bigg ( \ln \bigg ( \tau \bigg ) \bigg ) ^{2}lj{ I}\,\cosh \bigg ( 2\,\sqrt{-jl}\phi \bigg ) }{\sqrt{-jl}}}-\frac{1}{2} \,{\frac{ \bigg ( \ln \bigg ( \tau \bigg ) \bigg ) ^{2}lj{{ I}}^{2}\sinh \bigg ( 2\,\sqrt{-jl} \phi \bigg ) }{\sqrt{-jl}\cosh \bigg ( 2\,\sqrt{-jl}\phi \bigg ) }}+{ \frac{C\phi }{\sigma }} \bigg ),\\ \end{aligned} \end{aligned}$$

39 $$\begin{aligned}{} & {} \begin{aligned} u_{1,19}(x,t)&=e^{\left(\varrho B(t)-\frac{\varrho ^2 t}{2}\right)} \bigg ( 2\,\sigma \,\ln \bigg ( \tau \bigg ) rl-2\,\ln \bigg ( \tau \bigg ) l \sigma \, \bigg ( r-\sqrt{-{\frac{j}{l}}}\\&\quad \bigg ( \coth \bigg ( 2\, \sqrt{-jl}\phi \bigg ) +{ csch} \bigg ( 2\,\sqrt{-jl} \phi \bigg ) \bigg ) \bigg ) \bigg ),\\ z_{1,19}(x,t)&=e^{\left(\varrho B(t)-\frac{\varrho ^2 t}{2}\right)} \bigg ( - \bigg ( \ln \bigg ( \tau \bigg ) \bigg ) ^{2}lj\phi +\frac{1}{2} \,{\frac{ \bigg ( \ln \bigg ( \tau \bigg ) \bigg ) ^{2}lj\coth \bigg ( 2\,\sqrt{-jl}\phi \bigg ) }{\sqrt{-jl}}}\\&\quad +{\frac{ \bigg ( \ln \bigg ( \tau \bigg ) \bigg ) ^{2}lj \bigg ( \cosh \bigg ( 2\,\sqrt{-jl} \phi \bigg ) \bigg ) ^{2}}{\sqrt{-jl}\sinh \bigg ( 2\,\sqrt{-jl}\phi \bigg ) }}-{\frac{ \bigg ( \ln \bigg ( \tau \bigg ) \bigg ) ^{2}lj \sinh \bigg ( 2\,\sqrt{-jl}\phi \bigg ) }{\sqrt{-jl}}}\\&\quad +\frac{1}{2} \,{\frac{ \bigg ( \ln \bigg ( \tau \bigg ) \bigg ) ^{2}lj\cosh \bigg ( 2\,\sqrt{-jl}\phi \bigg ) }{\sqrt{-jl}\sinh \bigg ( 2\,\sqrt{-jl}\phi \bigg ) }}+{\frac{C\phi }{\sigma }} \bigg ),\\ \end{aligned} \end{aligned}$$

and

40 $$\begin{aligned} \begin{aligned} u_{1,20}(x,t)&=e^{\left(\varrho B(t)-\frac{\varrho ^2 t}{2}\right)} \bigg ( 2\,\sigma \,\ln \bigg ( \tau \bigg ) rl-2\,\ln \bigg ( \tau \bigg ) l \sigma \, \bigg ( r-\frac{1}{2} \,\sqrt{-{\frac{j}{l}}}\\&\quad \bigg ( \tanh \bigg ( \frac{1}{2} \,\sqrt{-jl}\phi \bigg ) +\coth \bigg ( \frac{1}{2} \,\sqrt{-jl}\phi \bigg ) \bigg ) \bigg ) \bigg ),\\ z_{1,20}(x,t)&=e^{\left(\varrho B(t)-\frac{\varrho ^2 t}{2}\right)} \bigg ( - \bigg ( \ln \bigg ( \tau \bigg ) \bigg ) ^{2}lj\phi +\frac{1}{2} \,{\frac{ \bigg ( \ln \bigg ( \tau \bigg ) \bigg ) ^{2}lj\tanh \bigg ( \frac{1}{2} \, \sqrt{-jl}\phi \bigg ) }{\sqrt{-jl}}}\\&\quad +\frac{1}{2} \,{\frac{ \bigg ( \ln \bigg ( \tau \bigg ) \bigg ) ^{2}lj\coth \bigg ( \frac{1}{2} \,\sqrt{-jl}\phi \bigg ) }{\sqrt{-jl}}}+{\frac{C\phi }{\sigma }} \bigg ).\\ \end{aligned} \end{aligned}$$

#### Family 1.5

When $$l=j$$ and $$k=0$$,

41 $$\begin{aligned}{} & {} \begin{aligned} u_{1,21}(x,t)&=e^{\left(\varrho B(t)-\frac{\varrho ^2 t}{2}\right)} \bigg ( 2\,\sigma \,\ln \bigg ( \tau \bigg ) rj-2\,\ln \bigg ( \tau \bigg ) j \sigma \, \bigg ( r+\tan \bigg ( j\phi \bigg ) \bigg ) \bigg ),\\ z_{1,21}(x,t)&=e^{\left(\varrho B(t)-\frac{\varrho ^2 t}{2}\right)} \bigg ( j \bigg ( \ln \bigg ( \tau \bigg ) \bigg ) ^{2}\tan \bigg ( j\phi \bigg ) -j \bigg ( \ln \bigg ( \tau \bigg ) \bigg ) ^{2}\arctan \bigg ( \tan \bigg ( j\phi \bigg ) \bigg ) +{\frac{C\phi }{\sigma }} \bigg ),\\ \end{aligned} \end{aligned}$$

42 $$\begin{aligned}{} & {} \begin{aligned} u_{1,22}(x,t)&=e^{\left(\varrho B(t)-\frac{\varrho ^2 t}{2}\right)} \bigg ( 2\,\sigma \,\ln \bigg ( \tau \bigg ) rj-2\,\ln \bigg ( \tau \bigg ) j \sigma \, \bigg ( r-\cot \bigg ( j\phi \bigg ) \bigg ) \bigg ),\\ z_{1,22}(x,t)&=e^{\left(\varrho B(t)-\frac{\varrho ^2 t}{2}\right)} \bigg ( -j \bigg ( \ln \bigg ( \tau \bigg ) \bigg ) ^{2}\cot \bigg ( j\phi \bigg ) \\&\quad +\frac{1}{2} \,j \bigg ( \ln \bigg ( \tau \bigg ) \bigg ) ^{2}\pi -j \bigg ( \ln \bigg ( \tau \bigg ) \bigg ) ^{2}{} { arccot} \bigg ( \cot \bigg ( j\phi \bigg ) \bigg ) +{\frac{C\phi }{\sigma }} \bigg ),\\ \end{aligned} \end{aligned}$$

43 $$\begin{aligned}{} & {} \begin{aligned} u_{1,23}(x,t)&=e^{\left(\varrho B(t)-\frac{\varrho ^2 t}{2}\right)} \bigg ( 2\,\sigma \,\ln \bigg ( \tau \bigg ) rj-2\,\ln \bigg ( \tau \bigg ) j \sigma \, \bigg ( r+\tan \bigg ( 2\,j\phi \bigg ) +\sec \bigg ( 2\,j\phi \bigg ) \bigg ) \bigg ),\\ z_{1,23}(x,t)&=e^{\left(\varrho B(t)-\frac{\varrho ^2 t}{2}\right)} \bigg ( \frac{1}{2} \,j \bigg ( \ln \bigg ( \tau \bigg ) \bigg ) ^{2}\tan \bigg ( 2\,j \phi \bigg ) - \bigg ( \ln \bigg ( \tau \bigg ) \bigg ) ^{2}{j}^{2} \phi +{\frac{j \bigg ( \ln \bigg ( \tau \bigg ) \bigg ) ^{2} }{\cos \bigg ( 2\,j\phi \bigg ) }}\\&\quad +\frac{1}{2} \,{\frac{j \bigg ( \ln \bigg ( \tau \bigg ) \bigg ) ^{2}\sin \bigg ( 2\,j\phi \bigg ) }{\cos \bigg ( 2\,j\phi \bigg ) }}+{\frac{C\phi }{\sigma }} \bigg ),\\ \end{aligned} \end{aligned}$$

44 $$\begin{aligned}{} & {} \begin{aligned} u_{1,24}(x,t)&=e^{\left(\varrho B(t)-\frac{\varrho ^2 t}{2}\right)} \bigg ( 2\,\sigma \,\ln \bigg ( \tau \bigg ) rj-2\,\ln \bigg ( \tau \bigg ) j \sigma \, \bigg ( r-\cot \bigg ( 2\,j\phi \bigg ) -\csc \bigg ( 2\,j\phi \bigg ) \bigg ) \bigg ),\\ z_{1,24}(x,t)&=e^{\left(\varrho B(t)-\frac{\varrho ^2 t}{2}\right)} \bigg ( -\frac{1}{2} \,j \bigg ( \ln \bigg ( \tau \bigg ) \bigg ) ^{2}\cot \bigg ( 2\,j \phi \bigg ) - \bigg ( \ln \bigg ( \tau \bigg ) \bigg ) ^{2}{j}^{2} \phi \\&\quad -{\frac{j \bigg ( \ln \bigg ( \tau \bigg ) \bigg ) ^{2} }{\sin \bigg ( 2\,j\phi \bigg ) }}-\frac{1}{2} \,{\frac{j \bigg ( \ln \bigg ( \tau \bigg ) \bigg ) ^{2}\cos \bigg ( 2\,j\phi \bigg ) }{\sin \bigg ( 2\,j\phi \bigg ) }}+{\frac{C\phi }{\sigma }} \bigg ),\\ \end{aligned} \end{aligned}$$

and

45 $$\begin{aligned} \begin{aligned} u_{1,25}(x,t)&=e^{\left(\varrho B(t)-\frac{\varrho ^2 t}{2}\right)} \bigg ( 2\,\sigma \,\ln \bigg ( \tau \bigg ) rj-2\,\ln \bigg ( \tau \bigg ) j \sigma \, \bigg ( r+\frac{1}{2} \,\tan \bigg ( \frac{1}{2} \,j\phi \bigg ) -\frac{1}{2} \,\cot \bigg ( \frac{1}{2} \,j\phi \bigg ) \bigg ) \bigg ),\\ z_{1,25}(x,t)&=e^{\left(\varrho B(t)-\frac{\varrho ^2 t}{2}\right)} \bigg ( \frac{1}{2} \,j \bigg ( \ln \bigg ( \tau \bigg ) \bigg ) ^{2}\tan \bigg ( \frac{1}{2} \,j \phi \bigg ) \\&\quad - \bigg ( \ln \bigg ( \tau \bigg ) \bigg ) ^{2}{j}^{2} \phi -\frac{1}{2} \,j \bigg ( \ln \bigg ( \tau \bigg ) \bigg ) ^{2}\cot \bigg ( 1 /2\,j\phi \bigg ) +{\frac{C\phi }{\sigma }} \bigg ).\\ \end{aligned} \end{aligned}$$

#### Family 1.6

When $$l=-j$$ and $$k=0$$,

46 $$\begin{aligned}{} & {} \begin{aligned} u_{1,26}(x,t)&=e^{\left(\varrho B(t)-\frac{\varrho ^2 t}{2}\right)} \bigg ( -2\,\sigma \,\ln \bigg ( \tau \bigg ) rj+2\,\ln \bigg ( \tau \bigg ) j \sigma \, \bigg ( r-\tanh \bigg ( j\phi \bigg ) \bigg ) \bigg ),\\ z_{1,26}(x,t)&=e^{\left(\varrho B(t)-\frac{\varrho ^2 t}{2}\right)} \bigg ( -j \bigg ( \ln \bigg ( \tau \bigg ) \bigg ) ^{2}\tanh \bigg ( j\phi \bigg ) -\frac{1}{2} \,j \bigg ( \ln \bigg ( \tau \bigg ) \bigg ) ^{2}\ln \bigg ( \tanh \bigg ( j\phi \bigg ) -1 \bigg )\\&\quad +\frac{1}{2} \,j \bigg ( \ln \bigg ( \tau \bigg ) \bigg ) ^{2}\ln \bigg ( \tanh \bigg ( j\phi \bigg ) +1 \bigg ) +{\frac{C\phi }{\sigma }} \bigg ),\\ \end{aligned} \end{aligned}$$

47 $$\begin{aligned}{} & {} \begin{aligned} u_{1,27}(x,t)&=e^{\left(\varrho B(t)-\frac{\varrho ^2 t}{2}\right)} \bigg ( -2\,\sigma \,\ln \bigg ( \tau \bigg ) rj+2\,\ln \bigg ( \tau \bigg ) j \sigma \, \bigg ( r-\coth \bigg ( j\phi \bigg ) \bigg ) \bigg ),\\ z_{1,27}(x,t)&=e^{\left(\varrho B(t)-\frac{\varrho ^2 t}{2}\right)} \bigg ( -j \bigg ( \ln \bigg ( \tau \bigg ) \bigg ) ^{2}\coth \bigg ( j\phi \bigg ) -\frac{1}{2} \,j \bigg ( \ln \bigg ( \tau \bigg ) \bigg ) ^{2}\ln \bigg ( \coth \bigg ( j\phi \bigg ) -1 \bigg )\\&\quad +\frac{1}{2} \,j \bigg ( \ln \bigg ( \tau \bigg ) \bigg ) ^{2}\ln \bigg ( \coth \bigg ( j\phi \bigg ) +1 \bigg ) +{\frac{C\phi }{\sigma }} \bigg ),\\ \end{aligned} \end{aligned}$$

48 $$\begin{aligned}{} & {} \begin{aligned} u_{1,28}(x,t)&=e^{\left(\varrho B(t)-\frac{\varrho ^2 t}{2}\right)} \bigg ( -2\,\sigma \,\ln \bigg ( \tau \bigg ) rj+2\,\ln \bigg ( \tau \bigg ) j \sigma \, \bigg ( r-\tanh \bigg ( 2\,j\phi \bigg ) -{ I}\,{ sech} \bigg ( 2\,j\phi \bigg ) \bigg ) \bigg ),\\ z_{1,28}(x,t)&=e^{\left(\varrho B(t)-\frac{\varrho ^2 t}{2}\right)} \bigg ( \bigg ( \ln \bigg ( \tau \bigg ) \bigg ) ^{2}{j}^{2}\phi -\frac{1}{2} \,j \bigg ( \ln \bigg ( \tau \bigg ) \bigg ) ^{2}\tanh \bigg ( 2\,j\phi \bigg ) \\&\quad +{\frac{j \bigg ( \ln \bigg ( \tau \bigg ) \bigg ) ^{2}{} { I}\, \bigg ( \sinh \bigg ( 2\,j\phi \bigg ) \bigg ) ^{2}}{ \cosh \bigg ( 2\,j\phi \bigg ) }}-j \bigg ( \ln \bigg ( \tau \bigg ) \bigg ) ^{2}{ I}\,\cosh \bigg ( 2\,j\phi \bigg ) \\&\quad +\frac{1}{2} \,{\frac{j \bigg ( \ln \bigg ( \tau \bigg ) \bigg ) ^{2}{{ I}}^ {2}\sinh \bigg ( 2\,j\phi \bigg ) }{\cosh \bigg ( 2\,j\phi \bigg ) }}+ {\frac{C\phi }{\sigma }} \bigg ),\\ \end{aligned} \end{aligned}$$

49 $$\begin{aligned}{} & {} \begin{aligned} u_{1,29}(x,t)&=e^{\left(\varrho B(t)-\frac{\varrho ^2 t}{2}\right)} \bigg ( -2\,\sigma \,\ln \bigg ( \tau \bigg ) rj+2\,\ln \bigg ( \tau \bigg ) j \sigma \, \bigg ( r-\coth \bigg ( 2\,j\phi \bigg ) -{ csch} \bigg ( 2\,j\phi \bigg ) \bigg ) \bigg ),\\ z_{1,29}(x,t)&=e^{\left(\varrho B(t)-\frac{\varrho ^2 t}{2}\right)} \bigg ( \bigg ( \ln \bigg ( \tau \bigg ) \bigg ) ^{2}{j}^{2}\phi -\frac{1}{2} \,j \bigg ( \ln \bigg ( \tau \bigg ) \bigg ) ^{2}\coth \bigg ( 2\,j\phi \bigg )\\&\quad -{\frac{j \bigg ( \ln \bigg ( \tau \bigg ) \bigg ) ^{2} \bigg ( \cosh \bigg ( 2\,j\phi \bigg ) \bigg ) ^{2}}{\sinh \bigg ( 2\,j\phi \bigg ) }}+j \bigg ( \ln \bigg ( \tau \bigg ) \bigg ) ^{2}\sinh \bigg ( 2\,j\phi \bigg ) \\&\quad -\frac{1}{2} \,{\frac{j \bigg ( \ln \bigg ( \tau \bigg ) \bigg ) ^{2}\cosh \bigg ( 2\,j\phi \bigg ) }{\sinh \bigg ( 2\,j\phi \bigg ) }}+{\frac{C\phi }{\sigma }} \bigg ),\\ \end{aligned} \end{aligned}$$

and

50 $$\begin{aligned} \begin{aligned} u_{1,30}(x,t)&=e^{\left(\varrho B(t)-\frac{\varrho ^2 t}{2}\right)} \bigg ( -2\,\sigma \,\ln \bigg ( \tau \bigg ) rj+2\,\ln \bigg ( \tau \bigg ) j \sigma \, \bigg ( r-\frac{1}{2} \,\tanh \bigg ( \frac{1}{2} \,j\phi \bigg ) -\frac{1}{2} \,\coth \bigg ( \frac{1}{2} \,j\phi \bigg ) \bigg ) \bigg ),\\ z_{1,30}(x,t)&=e^{\left(\varrho B(t)-\frac{\varrho ^2 t}{2}\right)} \bigg ( \bigg ( \ln \bigg ( \tau \bigg ) \bigg ) ^{2}{j}^{2}\phi -\frac{1}{2} \,j \bigg ( \ln \bigg ( \tau \bigg ) \bigg ) ^{2}\tanh \bigg ( \frac{1}{2} \,j\phi \bigg ) \\&\quad -\frac{1}{2} \,j \bigg ( \ln \bigg ( \tau \bigg ) \bigg ) ^{2}\coth \bigg ( \frac{1}{2} \,j\phi \bigg ) +{\frac{C\phi }{\sigma }} \bigg ).\\ \end{aligned} \end{aligned}$$

#### Family 1.7

When $$G=0$$,

51 $$\begin{aligned} \begin{aligned} u_{1,31}(x,t)&=e^{\left(\varrho B(t)-\frac{\varrho ^2 t}{2}\right)} \bigg ( {\frac{\sigma \, \bigg ( -{k}^{3}\phi \,\ln \bigg ( \tau \bigg ) +4\,ljk \phi \,\ln \bigg ( \tau \bigg ) +8\,lj \bigg ) }{{k}^{2}\phi }} \bigg ),\\ z_{1,31}(x,t)&=e^{\left(\varrho B(t)-\frac{\varrho ^2 t}{2}\right)} \bigg ( \frac{1}{4} \,{k}^{2} \bigg ( \ln \bigg ( \tau \bigg ) \bigg ) ^{2}\phi -2\, \bigg ( \ln \bigg ( \tau \bigg ) \bigg ) ^{2}\phi \,lj+4\,{\frac{ \bigg ( \ln \bigg ( \tau \bigg ) \bigg ) ^{2}\phi \,{l}^{2}{j}^{2}}{{k }^{2}}}\\&\quad -4\,{\frac{lj\ln \bigg ( \tau \bigg ) \ln \bigg ( \phi \bigg ) }{k}}+16\,{\frac{{l}^{2}{j}^{2}\ln \bigg ( \tau \bigg ) \ln \bigg ( \phi \bigg ) }{{k}^{3}}}-16\,{\frac{{l}^{2}{j}^{2}}{{k}^{4} \phi }}+{\frac{C\phi }{\sigma }} \bigg ).\\ \end{aligned} \end{aligned}$$

#### Family 1.8

When $$k=\eta$$, $$j=n\eta (n\ne 0)$$ and $$l=0$$,

52 $$\begin{aligned} u_{{1,32}} (x,t) = & e^{{\left( {{\varrho }B(t) - \frac{{{\varrho }^{2} t}}{2}} \right)}} ( - \sigma {\mkern 1mu} \ln (\tau )\eta ), \\ z_{{1,32}} (x,t) = & e^{{\left( {{\varrho }B(t) - \frac{{{\varrho }^{2} t}}{2}} \right)}} \left( {\frac{1}{4}{\mkern 1mu} \ln (\tau )\ln (\tau ^{{\eta {\kern 1pt} \phi }} )\eta + \frac{{C\phi }}{\sigma }} \right). \\ \end{aligned}$$

#### Family 1.9

When $$k=j=0$$,

53 $$\begin{aligned}&u_{1,33}(x,t)=e^{\left(\varrho B(t)-\frac{\varrho ^2 t}{2}\right)} \bigg ( 2\,{\frac{\sigma }{\phi }} \bigg ),\\&z_{1,33}(x,t)=e^{\left(\varrho B(t)-\frac{\varrho ^2 t}{2}\right)} \bigg ( -{\phi }^{-1}+{\frac{C\phi }{\sigma }} \bigg ).\\ \end{aligned}$$

#### Family 1.10

When $$k=\eta$$, $$l=n\eta (n\ne 0)$$ and $$j=0$$,

54 $$\begin{aligned} \begin{aligned} u_{1,34}(x,t)&=e^{\left(\varrho B(t)-\frac{\varrho ^2 t}{2}\right)} \bigg ( {\frac{\ln \bigg ( \tau \bigg ) \eta \,\sigma \, \bigg ( 1-n{\tau }^{\eta \,\phi }+2\,n{\tau }^{\eta \,\phi } \bigg ) }{-1+n{\tau }^{\eta \,\phi }}} \bigg ),\\ z_{1,34}(x,t)&=e^{\left(\varrho B(t)-\frac{\varrho ^2 t}{2}\right)} \bigg ( -\ln \bigg ( \tau \bigg ) \eta \,\ln \bigg ( -1+n{\tau }^{\eta \,\phi } \bigg ) +\ln \bigg ( \tau \bigg ) \eta \,\ln \bigg ( -1+n{\tau } ^{\eta \,\phi } \bigg )\\&\quad -{\frac{\ln \bigg ( \tau \bigg ) \eta \,}{-1+n{\tau }^{\eta \,\phi }}}+\frac{1}{4} \,\ln \bigg ( \tau \bigg ) \ln \bigg ( { \tau }^{\eta \,\phi } \bigg ) \eta +{\frac{C\phi }{\sigma }} \bigg ).\\ \end{aligned} \end{aligned}$$

Where $$\phi = \frac{\lambda x^\alpha }{\alpha }+\sigma t$$.

Considering case. 2 into (20), and utilizing (11), (16) and (18) together with the corresponding general solution of (10), we get the following families of stochastic soliton solutions for (1):

#### Family 2.1

When $$G<0 \quad l\ne 0$$,

55 $$\begin{aligned}{} & {} \begin{aligned} u_{2,1}(x,t)&=e^{\left(\varrho B(t)-\frac{\varrho ^2 t}{2}\right)} \bigg ( -k\ln \bigg ( \tau \bigg ) \bigg ( -2\,rl+k \bigg ) -2\,\ln \bigg ( \tau \bigg ) lk \\&\quad \bigg ( r-\frac{1}{2} \,{\frac{k}{l}}+\frac{1}{2} \,{\frac{\sqrt{-G} \tan \bigg ( \frac{1}{2} \,\sqrt{-G}\phi \bigg ) }{l}} \bigg ) \bigg ),\\ z_{2,1}(x,t)&=e^{\left(\varrho B(t)-\frac{\varrho ^2 t}{2}\right)} \bigg ( -\frac{1}{2} \,{\frac{ \bigg ( \ln \bigg ( \tau \bigg ) \bigg ) ^{2}G\tan \bigg ( \frac{1}{2} \,\sqrt{-G}\phi \bigg ) }{\sqrt{-G}}}\\&\quad +\frac{1}{2} \,{\frac{ \bigg ( \ln \bigg ( \tau \bigg ) \bigg ) ^{2}G\arctan \bigg ( \tan \bigg ( \frac{1}{2} \,\sqrt{-G}\phi \bigg ) \bigg ) }{\sqrt{-G}}}+{\frac{C \phi }{k}} \bigg ),\\ \end{aligned} \end{aligned}$$

56 $$\begin{aligned}{} & {} \begin{aligned} u_{2,2}(x,t)&=e^{\left(\varrho B(t)-\frac{\varrho ^2 t}{2}\right)} \bigg ( -k\ln \bigg ( \tau \bigg ) \bigg ( -2\,rl+k \bigg ) -2\,\ln \bigg ( \tau \bigg ) lk \bigg ( r-\frac{1}{2} \,{\frac{k}{l}}-\frac{1}{2} \,{\frac{\sqrt{-G} \cot \bigg ( \frac{1}{2} \,\sqrt{-G}\phi \bigg ) }{l}} \bigg ) \bigg ),\\ z_{2,2}(x,t)&=e^{\left(\varrho B(t)-\frac{\varrho ^2 t}{2}\right)} \bigg ( \frac{1}{2} \,{\frac{ \bigg ( \ln \bigg ( \tau \bigg ) \bigg ) ^{2}G\cot \bigg ( \frac{1}{2} \,\sqrt{-G}\phi \bigg ) }{\sqrt{-G}}}-\frac{1}{4} \,{\frac{ \bigg ( \ln \bigg ( \tau \bigg ) \bigg ) ^{2}G\pi }{\sqrt{-G}}}\\&\quad +\frac{1}{2} \,{\frac{ \bigg ( \ln \bigg ( \tau \bigg ) \bigg ) ^{2}G{ arccot} \bigg ( \cot \bigg ( \frac{1}{2} \,\sqrt{-G}\phi \bigg ) \bigg ) }{\sqrt{-G}} }+{\frac{C\phi }{k}} \bigg ),\\ \end{aligned} \end{aligned}$$

57 $$\begin{aligned}{} & {} \begin{aligned} u_{2,3}(x,t)&=e^{\left(\varrho B(t)-\frac{\varrho ^2 t}{2}\right)} \bigg ( -k\ln \bigg ( \tau \bigg ) \bigg ( -2\,rl+k \bigg ) -2\,\ln \bigg ( \tau \bigg ) lk \bigg ( r-\frac{1}{2} \,{\frac{k}{l}}\\&\quad +\frac{1}{2} \,{\frac{\sqrt{-G} \bigg ( \tan \bigg ( \sqrt{-G}\phi \bigg ) +\sec \bigg ( \sqrt{-G}\phi \bigg ) \bigg ) }{l}} \bigg ) \bigg ),\\ z_{2,3}(x,t)&=e^{\left(\varrho B(t)-\frac{\varrho ^2 t}{2}\right)} \bigg ( -\frac{1}{4} \,{\frac{ \bigg ( \ln \bigg ( \tau \bigg ) \bigg ) ^{2}G\tan \bigg ( \sqrt{-G}\phi \bigg ) }{\sqrt{-G}}}\\&\quad +\frac{1}{4} \, \bigg ( \ln \bigg ( \tau \bigg ) \bigg ) ^{2}G\phi -\frac{1}{2} \,{\frac{ \bigg ( \ln \bigg ( \tau \bigg ) \bigg ) ^{2}G}{\sqrt{-G}\cos \bigg ( \sqrt{-G}\phi \bigg ) }}\\&\quad -\frac{1}{4} \,{\frac{ \bigg ( \ln \bigg ( \tau \bigg ) \bigg ) ^{2}G\sin \bigg ( \sqrt{-G}\phi \bigg ) }{\sqrt{- G}\cos \bigg ( \sqrt{-G}\phi \bigg ) }}+{\frac{C\phi }{k}} \bigg ),\\ \end{aligned} \end{aligned}$$

58 $$\begin{aligned}{} & {} \begin{aligned} u_{2,4}(x,t)&=e^{\left(\varrho B(t)-\frac{\varrho ^2 t}{2}\right)} \bigg ( -k\ln \bigg ( \tau \bigg ) \bigg ( -2\,rl+k \bigg ) -2\,\ln \bigg ( \tau \bigg ) lk \bigg ( r-\frac{1}{2} \,{\frac{k}{l}}\\&\quad -\frac{1}{2} \,{\frac{\sqrt{-G} \bigg ( \cot \bigg ( \sqrt{-G}\phi \bigg ) +\csc \bigg ( \sqrt{-G}\phi \bigg ) \bigg ) }{l}} \bigg ) \bigg ),\\ z_{2,4}(x,t)&=e^{\left(\varrho B(t)-\frac{\varrho ^2 t}{2}\right)} \bigg ( \frac{1}{4} \,{\frac{ \bigg ( \ln \bigg ( \tau \bigg ) \bigg ) ^{2}G\cot \bigg ( \sqrt{-G}\phi \bigg ) }{\sqrt{-G}}}\\&\quad +\frac{1}{4} \, \bigg ( \ln \bigg ( \tau \bigg ) \bigg ) ^{2}G\phi +\frac{1}{2} \,{\frac{ \bigg ( \ln \bigg ( \tau \bigg ) \bigg ) ^{2}G}{\sqrt{-G}\sin \bigg ( \sqrt{-G}\phi \bigg ) }}\\&\quad +\frac{1}{4} \,{\frac{ \bigg ( \ln \bigg ( \tau \bigg ) \bigg ) ^{2}G\cos \bigg ( \sqrt{-G}\phi \bigg ) }{\sqrt{- G}\sin \bigg ( \sqrt{-G}\phi \bigg ) }}+{\frac{C\phi }{k}} \bigg ),\\ \end{aligned} \end{aligned}$$

and

59 $$\begin{aligned} \begin{aligned} u_{2,5}(x,t)&=e^{\left(\varrho B(t)-\frac{\varrho ^2 t}{2}\right)} \bigg ( -k\ln \bigg ( \tau \bigg ) \bigg ( -2\,rl+k \bigg ) -2\,\ln \bigg ( \tau \bigg ) lk \bigg ( r-\frac{1}{2} \,{\frac{k}{l}}\\&\quad +\frac{1}{4} \,{\frac{\sqrt{-G} \bigg ( \tan \bigg ( \frac{1}{4} \,\sqrt{-G}\phi \bigg ) -\cot \bigg ( \frac{1}{4} \, \sqrt{-G}\phi \bigg ) \bigg ) }{l}} \bigg ) \bigg ),\\ z_{2,5}(x,t)&=e^{\left(\varrho B(t)-\frac{\varrho ^2 t}{2}\right)} \bigg ( -\frac{1}{4} \,{\frac{ \bigg ( \ln \bigg ( \tau \bigg ) \bigg ) ^{2}G\tan \bigg ( \frac{1}{4} \,\sqrt{-G}\phi \bigg ) }{\sqrt{-G}}}\\&\quad +\frac{1}{4} \, \bigg ( \ln \bigg ( \tau \bigg ) \bigg ) ^{2}G\phi +\frac{1}{4} \,{\frac{ \bigg ( \ln \bigg ( \tau \bigg ) \bigg ) ^{2}G\cot \bigg ( \frac{1}{4} \,\sqrt{-G}\phi \bigg ) }{\sqrt{-G}}}+{\frac{C\phi }{k}} \bigg ).\\ \end{aligned} \end{aligned}$$

#### Family 2.2

When $$G>0 \quad l\ne 0$$,

60 $$\begin{aligned}{} & {} \begin{aligned} u_{2,6}(x,t)&=e^{\left(\varrho B(t)-\frac{\varrho ^2 t}{2}\right)} \bigg ( -k\ln \bigg ( \tau \bigg ) \bigg ( -2\,rl+k \bigg ) -2\,\ln \bigg ( \tau \bigg ) lk \\&\quad \bigg ( r-\frac{1}{2} \,{\frac{k}{l}}-\frac{1}{2} \,{\frac{\sqrt{G} \tanh \bigg ( \frac{1}{2} \,\sqrt{G}\phi \bigg ) }{l}} \bigg ) \bigg ),\\ z_{2,6}(x,t)&=e^{\left(\varrho B(t)-\frac{\varrho ^2 t}{2}\right)} \bigg ( -\frac{1}{2} \,\sqrt{G} \bigg ( \ln \bigg ( \tau \bigg ) \bigg ) ^{2}\tanh \bigg ( \frac{1}{2} \,\sqrt{G}\phi \bigg ) \\&\quad -\frac{1}{4} \,\sqrt{G} \bigg ( \ln \bigg ( \tau \bigg ) \bigg ) ^{2}\ln \bigg ( \tanh \bigg ( \frac{1}{2} \,\sqrt{G}\phi \bigg ) -1 \bigg )\\&\quad +\frac{1}{4} \,\sqrt{G} \bigg ( \ln \bigg ( \tau \bigg ) \bigg ) ^{2}\ln \bigg ( \tanh \bigg ( \frac{1}{2} \,\sqrt{G}\phi \bigg ) +1 \bigg ) +{\frac{C\phi }{k}} \bigg ),\\ \end{aligned} \end{aligned}$$

61 $$\begin{aligned}{} & {} \begin{aligned} u_{2,7}(x,t)&=e^{\left(\varrho B(t)-\frac{\varrho ^2 t}{2}\right)} \bigg ( -k\ln \bigg ( \tau \bigg ) \bigg ( -2\,rl+k \bigg ) -2\,\ln \bigg ( \tau \bigg ) lk\\&\quad \bigg ( r-\frac{1}{2} \,{\frac{k}{l}}-\frac{1}{2} \,{\frac{\sqrt{G} \coth \bigg ( \frac{1}{2} \,\sqrt{G}\phi \bigg ) }{l}} \bigg ) \bigg ),\\ z_{2,7}(x,t)&=e^{\left(\varrho B(t)-\frac{\varrho ^2 t}{2}\right)} \bigg ( -\frac{1}{2} \,\sqrt{G} \bigg ( \ln \bigg ( \tau \bigg ) \bigg ) ^{2}\coth \bigg ( \frac{1}{2} \,\sqrt{G}\phi \bigg ) \\&\quad -\frac{1}{4} \,\sqrt{G} \bigg ( \ln \bigg ( \tau \bigg ) \bigg ) ^{2}\ln \bigg ( \coth \bigg ( \frac{1}{2} \,\sqrt{G}\phi \bigg ) -1 \bigg ) \\&\quad +\frac{1}{4} \,\sqrt{G} \bigg ( \ln \bigg ( \tau \bigg ) \bigg ) ^{2}\ln \bigg ( \coth \bigg ( \frac{1}{2} \,\sqrt{G}\phi \bigg ) +1 \bigg ) +{\frac{C\phi }{k}} \bigg ),\\ \end{aligned} \end{aligned}$$

62 $$\begin{aligned}{} & {} \begin{aligned} u_{2,8}(x,t)&=e^{\left(\varrho B(t)-\frac{\varrho ^2 t}{2}\right)} \bigg ( -k\ln \bigg ( \tau \bigg ) \bigg ( -2\,rl+k \bigg ) -2\,\ln \bigg ( \tau \bigg ) lk \bigg ( r-\frac{1}{2} \,{\frac{k}{l}}\\&\quad -\frac{1}{2} \,{\frac{\sqrt{G} \bigg ( \tanh \bigg ( \sqrt{G}\phi \bigg ) +{ I}\,{ sech} \bigg ( \sqrt{G}\phi \bigg ) \bigg ) }{l}} \bigg ) \bigg ),\\ z_{2,8}(x,t)&=e^{\left(\varrho B(t)-\frac{\varrho ^2 t}{2}\right)} \bigg ( \frac{1}{4} \, \bigg ( \ln \bigg ( \tau \bigg ) \bigg ) ^{2}G\phi -\frac{1}{4} \,\sqrt{G } \bigg ( \ln \bigg ( \tau \bigg ) \bigg ) ^{2}\tanh \bigg ( \sqrt{G} \phi \bigg ) \\&\quad +\frac{1}{2} \,{\frac{\sqrt{G} \bigg ( \ln \bigg ( \tau \bigg ) \bigg ) ^{2}{} { I}\, \bigg ( \sinh \bigg ( \sqrt{G}\phi \bigg ) \bigg ) ^{2}}{\cosh \bigg ( \sqrt{G}\phi \bigg ) }}-\frac{1}{2} \, \sqrt{G} \bigg ( \ln \bigg ( \tau \bigg ) \bigg ) ^{2}{} { I}\, \cosh \bigg ( \sqrt{G}\phi \bigg ) \\&\quad +\frac{1}{4} \,{\frac{\sqrt{G} \bigg ( \ln \bigg ( \tau \bigg ) \bigg ) ^{2}{{ I}}^{2}\sinh \bigg ( \sqrt{G}\phi \bigg ) }{\cosh \bigg ( \sqrt{G}\phi \bigg ) }}+ {\frac{C\phi }{k}} \bigg ),\\ \end{aligned} \end{aligned}$$

63 $$\begin{aligned}{} & {} \begin{aligned} u_{2,9}(x,t)&=e^{\left(\varrho B(t)-\frac{\varrho ^2 t}{2}\right)} \bigg ( -k\ln \bigg ( \tau \bigg ) \bigg ( -2\,rl+k \bigg ) -2\,\ln \bigg ( \tau \bigg ) lk \bigg ( r-\frac{1}{2} \,{\frac{k}{l}}\\&\quad -\frac{1}{2} \,{\frac{\sqrt{G} \bigg ( \coth \bigg ( \sqrt{G}\phi \bigg ) +{ csch} \bigg ( \sqrt{G}\phi \bigg ) \bigg ) }{l}} \bigg ) \bigg ),\\ z_{2,9}(x,t)&=e^{\left(\varrho B(t)-\frac{\varrho ^2 t}{2}\right)} \bigg ( \frac{1}{4} \, \bigg ( \ln \bigg ( \tau \bigg ) \bigg ) ^{2}G\phi -\frac{1}{4} \,\sqrt{G } \bigg ( \ln \bigg ( \tau \bigg ) \bigg ) ^{2}\coth \bigg ( \sqrt{G} \phi \bigg )\\&\quad -\frac{1}{2} \,{\frac{\sqrt{G} \bigg ( \ln \bigg ( \tau \bigg ) \bigg ) ^{2} \bigg ( \cosh \bigg ( \sqrt{G}\phi \bigg ) \bigg ) ^{2}}{\sinh \bigg ( \sqrt{G}\phi \bigg ) }}+\frac{1}{2} \,\sqrt{G} \bigg ( \ln \bigg ( \tau \bigg ) \bigg ) ^{2}\sinh \bigg ( \sqrt{G}\phi \bigg )\\&\quad -\frac{1}{4} \,{\frac{\sqrt{G} \bigg ( \ln \bigg ( \tau \bigg ) \bigg ) ^{2}\cosh \bigg ( \sqrt{G}\phi \bigg ) }{\sinh \bigg ( \sqrt{G}\phi \bigg ) }}+{\frac{C\phi }{k}} \bigg ),\\ \end{aligned} \end{aligned}$$

and

64 $$\begin{aligned} \begin{aligned} u_{2,10}(x,t)&=e^{\left(\varrho B(t)-\frac{\varrho ^2 t}{2}\right)} \bigg ( -k\ln \bigg ( \tau \bigg ) \bigg ( -2\,rl+k \bigg ) -2\,\ln \bigg ( \tau \bigg ) lk \bigg ( r-\frac{1}{2} \,{\frac{k}{l}}\\&\quad -\frac{1}{4} \,{\frac{\sqrt{G} \bigg ( \tanh \bigg ( \frac{1}{4} \,\sqrt{G}\phi \bigg ) -\coth \bigg ( \frac{1}{4} \, \sqrt{G}\phi \bigg ) \bigg ) }{l}} \bigg ) \bigg ),\\ z_{2,10}(x,t)&=e^{\left(\varrho B(t)-\frac{\varrho ^2 t}{2}\right)} \bigg ( -\frac{1}{4} \,\sqrt{G} \bigg ( \ln \bigg ( \tau \bigg ) \bigg ) ^{2}\tanh \bigg ( \frac{1}{4} \,\sqrt{G}\phi \bigg ) \\&\quad -\frac{1}{4} \,\sqrt{G} \bigg ( \ln \bigg ( \tau \bigg ) \bigg ) ^{2}\coth \bigg ( \frac{1}{4} \,\sqrt{G}\phi \bigg ) +{ \frac{C\phi }{k}} \bigg ).\\ \end{aligned} \end{aligned}$$

#### Family 2.3

When $$lj>0$$ and $$k=0$$,

65 $$\begin{aligned}{} & {} \begin{aligned} u_{2,11}(x,t)&=e^{\left(\varrho B(t)-\frac{\varrho ^2 t}{2}\right)} \bigg ( 2\,\sigma \,\ln \bigg ( \tau \bigg ) rl-2\,\ln \bigg ( \tau \bigg ) l \sigma \, \bigg ( r+\sqrt{{\frac{j}{l}}}\tan \bigg ( \sqrt{jl}\phi \bigg ) \bigg ) \bigg ),\\ z_{2,11}(x,t)&=e^{\left(\varrho B(t)-\frac{\varrho ^2 t}{2}\right)} \bigg ( {\frac{ \bigg ( \ln \bigg ( \tau \bigg ) \bigg ) ^{2}lj\tan \bigg ( \sqrt{jl}\phi \bigg ) }{\sqrt{jl}}}-{\frac{ \bigg ( \ln \bigg ( \tau \bigg ) \bigg ) ^{2}\arctan \bigg ( \tan \bigg ( \sqrt{jl}\phi \bigg ) \bigg ) jl}{\sqrt{jl}}}+{\frac{C\phi }{\sigma }} \bigg ),\\ \end{aligned} \end{aligned}$$

66 $$\begin{aligned}{} & {} \begin{aligned} u_{2,12}(x,t)&=e^{\left(\varrho B(t)-\frac{\varrho ^2 t}{2}\right)} \bigg ( 2\,\sigma \,\ln \bigg ( \tau \bigg ) rl-2\,\ln \bigg ( \tau \bigg ) l \sigma \, \bigg ( r-\sqrt{{\frac{j}{l}}}\cot \bigg ( \sqrt{jl}\phi \bigg ) \bigg ) \bigg ),\\ z_{2,12}(x,t)&=e^{\left(\varrho B(t)-\frac{\varrho ^2 t}{2}\right)} \bigg ( -{\frac{ \bigg ( \ln \bigg ( \tau \bigg ) \bigg ) ^{2}lj\cot \bigg ( \sqrt{jl}\phi \bigg ) }{\sqrt{jl}}}\\&\quad +\frac{1}{2} \,{\frac{ \bigg ( \ln \bigg ( \tau \bigg ) \bigg ) ^{2}lj\pi }{\sqrt{jl}}}-{\frac{ \bigg ( \ln \bigg ( \tau \bigg ) \bigg ) ^{2}lj{ arccot} \bigg ( \cot \bigg ( \sqrt{jl}\phi \bigg ) \bigg ) }{\sqrt{jl}}}+{\frac{C \phi }{\sigma }} \bigg ),\\ \end{aligned} \end{aligned}$$

67 $$\begin{aligned}{} & {} \begin{aligned} u_{2,13}(x,t)&=e^{\left(\varrho B(t)-\frac{\varrho ^2 t}{2}\right)} \bigg ( 2\,\sigma \,\ln \bigg ( \tau \bigg ) rl-2\,\ln \bigg ( \tau \bigg ) l \sigma \, \bigg ( r+\sqrt{{\frac{j}{l}}} \bigg ( \tan \bigg ( 2\,\sqrt{ jl}\phi \bigg ) +\sec \bigg ( 2\,\sqrt{jl}\phi \bigg ) \bigg ) \bigg ) \bigg ),\\ z_{2,13}(x,t)&=e^{\left(\varrho B(t)-\frac{\varrho ^2 t}{2}\right)} \bigg ( \frac{1}{2} \,{\frac{ \bigg ( \ln \bigg ( \tau \bigg ) \bigg ) ^{2}lj\tan \bigg ( 2\,\sqrt{jl}\phi \bigg ) }{\sqrt{jl}}}- \bigg ( \ln \bigg ( \tau \bigg ) \bigg ) ^{2}lj\phi \\&\quad +{\frac{ \bigg ( \ln \bigg ( \tau \bigg ) \bigg ) ^{2}lj}{\sqrt{jl}\cos \bigg ( 2\,\sqrt{jl }\phi \bigg ) }}+\frac{1}{2} \,{\frac{ \bigg ( \ln \bigg ( \tau \bigg ) \bigg ) ^{2}lj\sin \bigg ( 2\,\sqrt{jl}\phi \bigg ) }{\sqrt{jl} \cos \bigg ( 2\,\sqrt{jl}\phi \bigg ) }}+{\frac{C\phi }{\sigma }} \bigg ),\\ \end{aligned} \end{aligned}$$

68 $$\begin{aligned}{} & {} \begin{aligned} u_{2,14}(x,t)&=e^{\left(\varrho B(t)-\frac{\varrho ^2 t}{2}\right)} \bigg ( 2\,\sigma \,\ln \bigg ( \tau \bigg ) rl-2\,\ln \bigg ( \tau \bigg ) l \sigma \, \bigg ( r-\sqrt{{\frac{j}{l}}} \bigg ( \cot \bigg ( 2\,\sqrt{ jl}\phi \bigg ) +\csc \bigg ( 2\,\sqrt{jl}\phi \bigg ) \bigg ) \bigg ) \bigg ),\\ z_{2,14}(x,t)&=e^{\left(\varrho B(t)-\frac{\varrho ^2 t}{2}\right)} \bigg ( -\frac{1}{2} \,{\frac{ \bigg ( \ln \bigg ( \tau \bigg ) \bigg ) ^{2}lj\cot \bigg ( 2\,\sqrt{jl}\phi \bigg ) }{\sqrt{jl}}}- \bigg ( \ln \bigg ( \tau \bigg ) \bigg ) ^{2}lj\phi \\&\quad -{\frac{ \bigg ( \ln \bigg ( \tau \bigg ) \bigg ) ^{2}lj}{\sqrt{jl}\sin \bigg ( 2\,\sqrt{jl }\phi \bigg ) }}-\frac{1}{2} \,{\frac{ \bigg ( \ln \bigg ( \tau \bigg ) \bigg ) ^{2}lj\cos \bigg ( 2\,\sqrt{jl}\phi \bigg ) }{\sqrt{jl} \sin \bigg ( 2\,\sqrt{jl}\phi \bigg ) }}+{\frac{C\phi }{\sigma }} \bigg ),\\ \end{aligned} \end{aligned}$$

and

69 $$\begin{aligned} \begin{aligned} u_{2,15}(x,t)&=e^{\left(\varrho B(t)-\frac{\varrho ^2 t}{2}\right)} \bigg ( 2\,\sigma \,\ln \bigg ( \tau \bigg ) rl-2\,\ln \bigg ( \tau \bigg ) l \sigma \, \bigg ( r+\frac{1}{2} \,\sqrt{{\frac{j}{l}}} \bigg ( \tan \bigg ( \frac{1}{2} \, \sqrt{jl}\phi \bigg ) -\cot \bigg ( \frac{1}{2} \,\sqrt{jl}\phi \bigg ) \bigg ) \bigg ) \bigg ),\\ z_{2,15}(x,t)&=e^{\left(\varrho B(t)-\frac{\varrho ^2 t}{2}\right)} \bigg ( \frac{1}{2} \,{\frac{ \bigg ( \ln \bigg ( \tau \bigg ) \bigg ) ^{2}lj\tan \bigg ( \frac{1}{2} \,\sqrt{jl}\phi \bigg ) }{\sqrt{jl}}}\\&\quad - \bigg ( \ln \bigg ( \tau \bigg ) \bigg ) ^{2}lj\phi -\frac{1}{2} \,{\frac{ \bigg ( \ln \bigg ( \tau \bigg ) \bigg ) ^{2}lj\cot \bigg ( \frac{1}{2} \,\sqrt{jl}\phi \bigg ) }{\sqrt{jl}}}+{\frac{C\phi }{\sigma }} \bigg ).\\ \end{aligned} \end{aligned}$$

#### Family 2.4

When $$lj>0$$ and $$k=0$$,

70 $$\begin{aligned}{} & {} \begin{aligned} u_{2,16}(x,t)&=e^{\left(\varrho B(t)-\frac{\varrho ^2 t}{2}\right)} \bigg ( 2\,\sigma \,\ln \bigg ( \tau \bigg ) rl-2\,\ln \bigg ( \tau \bigg ) l \sigma \, \bigg ( r-\sqrt{-{\frac{j}{l}}}\tanh \bigg ( \sqrt{-jl}\phi \bigg ) \bigg ) \bigg ),\\ z_{2,16}(x,t)&=e^{\left(\varrho B(t)-\frac{\varrho ^2 t}{2}\right)} \bigg ( {\frac{ \bigg ( \ln \bigg ( \tau \bigg ) \bigg ) ^{2}lj\tanh \bigg ( \sqrt{-jl}\phi \bigg ) }{\sqrt{-jl}}}+\frac{1}{2} \,{\frac{ \bigg ( \ln \bigg ( \tau \bigg ) \bigg ) ^{2}\ln \bigg ( \tanh \bigg ( \sqrt{-jl} \phi \bigg ) -1 \bigg ) jl}{\sqrt{-jl}}}\\&\quad -\frac{1}{2} \,{\frac{ \bigg ( \ln \bigg ( \tau \bigg ) \bigg ) ^{2}\ln \bigg ( \tanh \bigg ( \sqrt{-jl} \phi \bigg ) +1 \bigg ) jl}{\sqrt{-jl}}}+{\frac{C\phi }{\sigma }} \bigg ),\\ \end{aligned} \end{aligned}$$

71 $$\begin{aligned}{} & {} \begin{aligned} u_{2,17}(x,t)&=e^{\left(\varrho B(t)-\frac{\varrho ^2 t}{2}\right)} \bigg ( 2\,\sigma \,\ln \bigg ( \tau \bigg ) rl-2\,\ln \bigg ( \tau \bigg ) l \sigma \, \bigg ( r-\sqrt{-{\frac{j}{l}}}\coth \bigg ( \sqrt{-jl}\phi \bigg ) \bigg ) \bigg ),\\ z_{2,17}(x,t)&=e^{\left(\varrho B(t)-\frac{\varrho ^2 t}{2}\right)} \bigg ( {\frac{ \bigg ( \ln \bigg ( \tau \bigg ) \bigg ) ^{2}lj\coth \bigg ( \sqrt{-jl}\phi \bigg ) }{\sqrt{-jl}}}+\frac{1}{2} \,{\frac{ \bigg ( \ln \bigg ( \tau \bigg ) \bigg ) ^{2}\ln \bigg ( \coth \bigg ( \sqrt{-jl} \phi \bigg ) -1 \bigg ) jl}{\sqrt{-jl}}}\\&\quad -\frac{1}{2} \,{\frac{ \bigg ( \ln \bigg ( \tau \bigg ) \bigg ) ^{2}\ln \bigg ( \coth \bigg ( \sqrt{-jl} \phi \bigg ) +1 \bigg ) jl}{\sqrt{-jl}}}+{\frac{C\phi }{\sigma }} \bigg ),\\ \end{aligned} \end{aligned}$$

72 $$\begin{aligned}{} & {} \begin{aligned} u_{2,18}(x,t)&=e^{\left(\varrho B(t)-\frac{\varrho ^2 t}{2}\right)} \bigg ( 2\,\sigma \,\ln \bigg ( \tau \bigg ) rl-2\,\ln \bigg ( \tau \bigg ) l\, \sigma \, \bigg ( r\\&\quad -\sqrt{-{\frac{j}{l}}} \bigg ( \tanh \bigg ( 2\, \sqrt{-jl}\phi \bigg ) +{ I}\,{ sech} \bigg ( 2\, \sqrt{-jl}\phi \bigg ) \bigg ) \bigg ) \bigg ),\\ z_{2,18}(x,t)&=e^{\left(\varrho B(t)-\frac{\varrho ^2 t}{2}\right)} \bigg ( - \bigg ( \ln \bigg ( \tau \bigg ) \bigg ) ^{2}lj\phi +\frac{1}{2} \,{\frac{ \bigg ( \ln \bigg ( \tau \bigg ) \bigg ) ^{2}lj\tanh \bigg ( 2\,\sqrt{-jl}\phi \bigg ) }{\sqrt{-jl}}}\\&\quad -{\frac{ \bigg ( \ln \bigg ( \tau \bigg ) \bigg ) ^{2}lj{ I}\, \bigg ( \sinh \bigg ( 2\, \sqrt{-jl}\phi \bigg ) \bigg ) ^{2}}{\sqrt{-jl}\cosh \bigg ( 2\, \sqrt{-jl}\phi \bigg ) }}\\&\quad +{\frac{ \bigg ( \ln \bigg ( \tau \bigg ) \bigg ) ^{2}lj{ I}\,\cosh \bigg ( 2\,\sqrt{-jl}\phi \bigg ) }{\sqrt{-jl}}}-\frac{1}{2} \,{\frac{ \bigg ( \ln \bigg ( \tau \bigg ) \bigg ) ^{2}lj{{ I}}^{2}\sinh \bigg ( 2\,\sqrt{-jl} \phi \bigg ) }{\sqrt{-jl}\cosh \bigg ( 2\,\sqrt{-jl}\phi \bigg ) }}+{ \frac{C\phi }{\sigma }} \bigg ),\\ \end{aligned} \end{aligned}$$

73 $$\begin{aligned}{} & {} \begin{aligned} u_{2,19}(x,t)&=e^{\left(\varrho B(t)-\frac{\varrho ^2 t}{2}\right)} \bigg ( 2\,\sigma \,\ln \bigg ( \tau \bigg ) rl-2\,\ln \bigg ( \tau \bigg ) l \,\sigma \, \bigg ( r\\&-\sqrt{-{\frac{j}{l}}} \bigg ( \coth \bigg ( 2\, \sqrt{-jl}\phi \bigg ) +{ csch} \bigg ( 2\,\sqrt{-jl} \phi \bigg ) \bigg ) \bigg ) \bigg ),\\ z_{2,19}(x,t)&=e^{\left(\varrho B(t)-\frac{\varrho ^2 t}{2}\right)} \bigg ( - \bigg ( \ln \bigg ( \tau \bigg ) \bigg ) ^{2}lj\phi +\frac{1}{2} \,{\frac{ \bigg ( \ln \bigg ( \tau \bigg ) \bigg ) ^{2}lj\coth \bigg ( 2\,\sqrt{-jl}\phi \bigg ) }{\sqrt{-jl}}}\\&\quad +{\frac{ \bigg ( \ln \bigg ( \tau \bigg ) \bigg ) ^{2}lj \bigg ( \cosh \bigg ( 2\,\sqrt{-jl} \phi \bigg ) \bigg ) ^{2}}{\sqrt{-jl}\sinh \bigg ( 2\,\sqrt{-jl}\phi \bigg ) }}-{\frac{ \bigg ( \ln \bigg ( \tau \bigg ) \bigg ) ^{2}lj \sinh \bigg ( 2\,\sqrt{-jl}\phi \bigg ) }{\sqrt{-jl}}}\\&\quad +\frac{1}{2} \,{\frac{ \bigg ( \ln \bigg ( \tau \bigg ) \bigg ) ^{2}lj\cosh \bigg ( 2\,\sqrt{-jl}\phi \bigg ) }{\sqrt{-jl}\sinh \bigg ( 2\,\sqrt{-jl}\phi \bigg ) }}+{\frac{C\phi }{\sigma }} \bigg ),\\ \end{aligned} \end{aligned}$$

and

74 $$\begin{aligned} \begin{aligned} u_{2,20}(x,t)&=e^{\left(\varrho B(t)-\frac{\varrho ^2 t}{2}\right)} \bigg ( 2\,\sigma \,\ln \bigg ( \tau \bigg ) rl-2\,\ln \bigg ( \tau \bigg ) l\, \sigma \, \bigg ( r\\&\quad -\frac{1}{2} \,\sqrt{-{\frac{j}{l}}} \bigg ( \tanh \bigg ( \frac{1}{2} \,\sqrt{-jl}\phi \bigg ) +\coth \bigg ( \frac{1}{2} \,\sqrt{-jl}\phi \bigg ) \bigg ) \bigg ) \bigg ),\\ z_{2,20}(x,t)&=e^{\left(\varrho B(t)-\frac{\varrho ^2 t}{2}\right)} \bigg ( - \bigg ( \ln \bigg ( \tau \bigg ) \bigg ) ^{2}lj\phi +\frac{1}{2} \,{\frac{ \bigg ( \ln \bigg ( \tau \bigg ) \bigg ) ^{2}lj\tanh \bigg ( \frac{1}{2} \, \sqrt{-jl}\phi \bigg ) }{\sqrt{-jl}}}\\&\quad +\frac{1}{2} \,{\frac{ \bigg ( \ln \bigg ( \tau \bigg ) \bigg ) ^{2}lj\coth \bigg ( \frac{1}{2} \,\sqrt{-jl}\phi \bigg ) }{\sqrt{-jl}}}+{\frac{C\phi }{\sigma }} \bigg ).\\ \end{aligned} \end{aligned}$$

#### Family 2.5

When $$l=j$$ and $$k=0$$,

75 $$\begin{aligned}{} & {} \begin{aligned} u_{2,21}(x,t)&=e^{\left(\varrho B(t)-\frac{\varrho ^2 t}{2}\right)} \bigg ( 2\,\sigma \,\ln \bigg ( \tau \bigg ) rj-2\,\ln \bigg ( \tau \bigg ) j \sigma \, \bigg ( r+\tan \bigg ( j\phi \bigg ) \bigg ) \bigg ),\\ z_{2,21}(x,t)&=e^{\left(\varrho B(t)-\frac{\varrho ^2 t}{2}\right)} \bigg ( j \bigg ( \ln \bigg ( \tau \bigg ) \bigg ) ^{2}\tan \bigg ( j\phi \bigg ) -j \bigg ( \ln \bigg ( \tau \bigg ) \bigg ) ^{2}\arctan \bigg ( \tan \bigg ( j\phi \bigg ) \bigg ) +{\frac{C\phi }{\sigma }} \bigg ),\\ \end{aligned} \end{aligned}$$

76 $$\begin{aligned}{} & {} \begin{aligned} u_{2,22}(x,t)&=e^{\left(\varrho B(t)-\frac{\varrho ^2 t}{2}\right)} \bigg ( 2\,\sigma \,\ln \bigg ( \tau \bigg ) rj-2\,\ln \bigg ( \tau \bigg ) j \sigma \, \bigg ( r-\cot \bigg ( j\phi \bigg ) \bigg ) \bigg ),\\ z_{2,22}(x,t)&=e^{\left(\varrho B(t)-\frac{\varrho ^2 t}{2}\right)} \bigg ( -j \bigg ( \ln \bigg ( \tau \bigg ) \bigg ) ^{2}\cot \bigg ( j\phi \bigg )\\&\quad +\frac{1}{2} \,j \bigg ( \ln \bigg ( \tau \bigg ) \bigg ) ^{2}\pi -j \bigg ( \ln \bigg ( \tau \bigg ) \bigg ) ^{2}{} { arccot} \bigg ( \cot \bigg ( j\phi \bigg ) \bigg ) +{\frac{C\phi }{\sigma }} \bigg ),\\ \end{aligned} \end{aligned}$$

77 $$\begin{aligned}{} & {} \begin{aligned} u_{2,23}(x,t)&=e^{\left(\varrho B(t)-\frac{\varrho ^2 t}{2}\right)} \bigg ( 2\,\sigma \,\ln \bigg ( \tau \bigg ) rj-2\,\ln \bigg ( \tau \bigg ) j \sigma \, \bigg ( r+\tan \bigg ( 2\,j\phi \bigg ) +\sec \bigg ( 2\,j\phi \bigg ) \bigg ) \bigg ),\\ z_{2,23}(x,t)&=e^{\left(\varrho B(t)-\frac{\varrho ^2 t}{2}\right)} \bigg ( \frac{1}{2} \,j \bigg ( \ln \bigg ( \tau \bigg ) \bigg ) ^{2}\tan \bigg ( 2\,j \phi \bigg ) - \bigg ( \ln \bigg ( \tau \bigg ) \bigg ) ^{2}{j}^{2} \phi \\&\quad +{\frac{j \bigg ( \ln \bigg ( \tau \bigg ) \bigg ) ^{2} }{\cos \bigg ( 2\,j\phi \bigg ) }}+\frac{1}{2} \,{\frac{j \bigg ( \ln \bigg ( \tau \bigg ) \bigg ) ^{2}\sin \bigg ( 2\,j\phi \bigg ) }{\cos \bigg ( 2\,j\phi \bigg ) }}+{\frac{C\phi }{\sigma }} \bigg ),\\ \end{aligned} \end{aligned}$$

78 $$\begin{aligned}{} & {} \begin{aligned} u_{2,24}(x,t)&=e^{\left(\varrho B(t)-\frac{\varrho ^2 t}{2}\right)} \bigg ( 2\,\sigma \,\ln \bigg ( \tau \bigg ) rj-2\,\ln \bigg ( \tau \bigg ) j \sigma \, \bigg ( r-\cot \bigg ( 2\,j\phi \bigg ) -\csc \bigg ( 2\,j\phi \bigg ) \bigg ) \bigg ),\\ z_{2,24}(x,t)&=e^{\left(\varrho B(t)-\frac{\varrho ^2 t}{2}\right)} \bigg ( -\frac{1}{2} \,j \bigg ( \ln \bigg ( \tau \bigg ) \bigg ) ^{2}\cot \bigg ( 2\,j \phi \bigg ) - \bigg ( \ln \bigg ( \tau \bigg ) \bigg ) ^{2}{j}^{2} \phi \\&\quad -{\frac{j \bigg ( \ln \bigg ( \tau \bigg ) \bigg ) ^{2} }{\sin \bigg ( 2\,j\phi \bigg ) }}-\frac{1}{2} \,{\frac{j \bigg ( \ln \bigg ( \tau \bigg ) \bigg ) ^{2}\cos \bigg ( 2\,j\phi \bigg ) }{\sin \bigg ( 2\,j\phi \bigg ) }}+{\frac{C\phi }{\sigma }} \bigg ),\\ \end{aligned} \end{aligned}$$

and

79 $$\begin{aligned} \begin{aligned} u_{2,25}(x,t)&=e^{\left(\varrho B(t)-\frac{\varrho ^2 t}{2}\right)} \bigg ( 2\,\sigma \,\ln \bigg ( \tau \bigg ) rj-2\,\ln \bigg ( \tau \bigg ) j \sigma \, \bigg ( r+\frac{1}{2} \,\tan \bigg ( \frac{1}{2} \,j\phi \bigg ) -\frac{1}{2} \,\cot \bigg ( \frac{1}{2} \,j\phi \bigg ) \bigg ) \bigg ),\\ z_{2,25}(x,t)&=e^{\left(\varrho B(t)-\frac{\varrho ^2 t}{2}\right)} \bigg ( \frac{1}{2} \,j \bigg ( \ln \bigg ( \tau \bigg ) \bigg ) ^{2}\tan \bigg ( \frac{1}{2} \,j \phi \bigg ) - \bigg ( \ln \bigg ( \tau \bigg ) \bigg ) ^{2}{j}^{2} \phi \\&\quad -\frac{1}{2} \,j \bigg ( \ln \bigg ( \tau \bigg ) \bigg ) ^{2}\cot \bigg ( 1 /2\,j\phi \bigg ) +{\frac{C\phi }{\sigma }} \bigg ).\\ \end{aligned} \end{aligned}$$

#### Family 2.6

When $$l=-j$$ and $$k=0$$,

80 $$\begin{aligned}{} & {} \begin{aligned} u_{2,26}(x,t)&=e^{\left(\varrho B(t)-\frac{\varrho ^2 t}{2}\right)} \bigg ( -2\,\sigma \,\ln \bigg ( \tau \bigg ) rj+2\,\ln \bigg ( \tau \bigg ) j \sigma \, \bigg ( r-\tanh \bigg ( j\phi \bigg ) \bigg ) \bigg ),\\ z_{2,26}(x,t)&=e^{\left(\varrho B(t)-\frac{\varrho ^2 t}{2}\right)} \bigg ( -j \bigg ( \ln \bigg ( \tau \bigg ) \bigg ) ^{2}\tanh \bigg ( j\phi \bigg ) -\frac{1}{2} \,j \bigg ( \ln \bigg ( \tau \bigg ) \bigg ) ^{2}\ln \bigg ( \tanh \bigg ( j\phi \bigg ) -1 \bigg ) \\&\quad +\frac{1}{2} \,j \bigg ( \ln \bigg ( \tau \bigg ) \bigg ) ^{2}\ln \bigg ( \tanh \bigg ( j\phi \bigg ) +1 \bigg ) +{\frac{C\phi }{\sigma }} \bigg ),\\ \end{aligned} \end{aligned}$$

81 $$\begin{aligned}{} & {} \begin{aligned} u_{2,27}(x,t)&=e^{\left(\varrho B(t)-\frac{\varrho ^2 t}{2}\right)} \bigg ( -2\,\sigma \,\ln \bigg ( \tau \bigg ) rj+2\,\ln \bigg ( \tau \bigg ) j \sigma \, \bigg ( r-\coth \bigg ( j\phi \bigg ) \bigg ) \bigg ),\\ z_{2,27}(x,t)&=e^{\left(\varrho B(t)-\frac{\varrho ^2 t}{2}\right)} \bigg ( -j \bigg ( \ln \bigg ( \tau \bigg ) \bigg ) ^{2}\coth \bigg ( j\phi \bigg ) -\frac{1}{2} \,j \bigg ( \ln \bigg ( \tau \bigg ) \bigg ) ^{2}\ln \bigg ( \coth \bigg ( j\phi \bigg ) -1 \bigg ) \\&\quad +\frac{1}{2} \,j \bigg ( \ln \bigg ( \tau \bigg ) \bigg ) ^{2}\ln \bigg ( \coth \bigg ( j\phi \bigg ) +1 \bigg ) +{\frac{C\phi }{\sigma }} \bigg ),\\ \end{aligned} \end{aligned}$$

82 $$\begin{aligned}{} & {} \begin{aligned} u_{2,28}(x,t)&=e^{\left(\varrho B(t)-\frac{\varrho ^2 t}{2}\right)} \bigg ( -2\,\sigma \,\ln \bigg ( \tau \bigg ) rj+2\,\ln \bigg ( \tau \bigg ) j \sigma \, \bigg ( r-\tanh \bigg ( 2\,j\phi \bigg ) -{ I}\,{ sech} \bigg ( 2\,j\phi \bigg ) \bigg ) \bigg ),\\ z_{2,28}(x,t)&=e^{\left(\varrho B(t)-\frac{\varrho ^2 t}{2}\right)} \bigg ( \bigg ( \ln \bigg ( \tau \bigg ) \bigg ) ^{2}{j}^{2}\phi -\frac{1}{2} \,j \bigg ( \ln \bigg ( \tau \bigg ) \bigg ) ^{2}\tanh \bigg ( 2\,j\phi \bigg )\\&\quad +{\frac{j \bigg ( \ln \bigg ( \tau \bigg ) \bigg ) ^{2}{} { I}\, \bigg ( \sinh \bigg ( 2\,j\phi \bigg ) \bigg ) ^{2}}{ \cosh \bigg ( 2\,j\phi \bigg ) }}-j \bigg ( \ln \bigg ( \tau \bigg ) \bigg ) ^{2}{ I}\,\cosh \bigg ( 2\,j\phi \bigg ) \\&\quad +\frac{1}{2} \,{\frac{j \bigg ( \ln \bigg ( \tau \bigg ) \bigg ) ^{2}{{ I}}^ {2}\sinh \bigg ( 2\,j\phi \bigg ) }{\cosh \bigg ( 2\,j\phi \bigg ) }}+ {\frac{C\phi }{\sigma }} \bigg ),\\ \end{aligned} \end{aligned}$$

83 $$\begin{aligned}{} & {} \begin{aligned} u_{2,29}(x,t)&=e^{\left(\varrho B(t)-\frac{\varrho ^2 t}{2}\right)} \bigg ( -2\,\sigma \,\ln \bigg ( \tau \bigg ) rj+2\,\ln \bigg ( \tau \bigg ) j \sigma \, \bigg ( r-\coth \bigg ( 2\,j\phi \bigg ) -{ csch} \bigg ( 2\,j\phi \bigg ) \bigg ) \bigg ),\\ z_{2,29}(x,t)&=e^{\left(\varrho B(t)-\frac{\varrho ^2 t}{2}\right)} \bigg ( \bigg ( \ln \bigg ( \tau \bigg ) \bigg ) ^{2}{j}^{2}\phi -\frac{1}{2} \,j \bigg ( \ln \bigg ( \tau \bigg ) \bigg ) ^{2}\coth \bigg ( 2\,j\phi \bigg ) \\&\quad -{\frac{j \bigg ( \ln \bigg ( \tau \bigg ) \bigg ) ^{2} \bigg ( \cosh \bigg ( 2\,j\phi \bigg ) \bigg ) ^{2}}{\sinh \bigg ( 2\,j\phi \bigg ) }}+j \bigg ( \ln \bigg ( \tau \bigg ) \bigg ) ^{2}\sinh \bigg ( 2\,j\phi \bigg ) \\&\quad -\frac{1}{2} \,{\frac{j \bigg ( \ln \bigg ( \tau \bigg ) \bigg ) ^{2}\cosh \bigg ( 2\,j\phi \bigg ) }{\sinh \bigg ( 2\,j\phi \bigg ) }}+{\frac{C\phi }{\sigma }} \bigg ),\\ \end{aligned} \end{aligned}$$

and

84 $$\begin{aligned} \begin{aligned} u_{2,30}(x,t)&=e^{\left(\varrho B(t)-\frac{\varrho ^2 t}{2}\right)} \bigg ( -2\,\sigma \,\ln \bigg ( \tau \bigg ) rj+2\,\ln \bigg ( \tau \bigg ) j \sigma \, \bigg ( r-\frac{1}{2} \,\tanh \bigg ( \frac{1}{2} \,j\phi \bigg ) -\frac{1}{2} \,\coth \bigg ( \frac{1}{2} \,j\phi \bigg ) \bigg ) \bigg ),\\ z_{2,30}(x,t)&=e^{\left(\varrho B(t)-\frac{\varrho ^2 t}{2}\right)} \bigg ( \bigg ( \ln \bigg ( \tau \bigg ) \bigg ) ^{2}{j}^{2}\phi -\frac{1}{2} \,j \bigg ( \ln \bigg ( \tau \bigg ) \bigg ) ^{2}\tanh \bigg ( \frac{1}{2} \,j\phi \bigg ) \\&\quad -\frac{1}{2} \,j \bigg ( \ln \bigg ( \tau \bigg ) \bigg ) ^{2}\coth \bigg ( \frac{1}{2} \,j\phi \bigg ) +{\frac{C\phi }{\sigma }} \bigg ).\\ \end{aligned} \end{aligned}$$

#### Family 2.8

When $$k=\eta$$, $$j=n\eta (n\ne 0)$$ and $$l=0$$,

85 $$\begin{aligned} &u_{2,32}(x,t)=e^{\left(\varrho B(t)-\frac{\varrho ^2 t}{2}\right)} \bigg ( I\sqrt{2C} \bigg ),\\&z_{2,32}(x,t)=e^{\left(\varrho B(t)-\frac{\varrho ^2 t}{2}\right)} \bigg ( -\frac{1}{2} \,{\frac{C\ln \bigg ( {\tau }^{\eta \,\phi } \bigg ) }{{\sigma }^{2} \eta \,\ln \bigg ( \tau \bigg ) }}+{\frac{C\phi }{\sigma }} \bigg ).\\ \end{aligned}$$

#### Family 2.9

When $$k=\eta$$, $$l=n\eta (n\ne 0)$$ and $$j=0$$,

86 $$\begin{aligned} \begin{aligned} u_{2,33}(x,t)&=e^{\left(\varrho B(t)-\frac{\varrho ^2 t}{2}\right)} \bigg ( -{\frac{\sqrt{-2\,C} \bigg ( 1-n{\tau }^{\eta \,\phi }+2\,n{\tau }^{\eta \,\phi } \bigg ) }{-1+n{\tau }^{\eta \,\phi }}} \bigg ),\\ z_{2,33}(x,t)&=e^{\left(\varrho B(t)-\frac{\varrho ^2 t}{2}\right)} \bigg ( 2\,{\frac{C\ln \bigg ( -1+n{\tau }^{\eta \,\phi } \bigg ) }{{\sigma }^{2 }\eta \,\ln \bigg ( \tau \bigg ) }}-2\,{\frac{C\ln \bigg ( -1+n {\tau }^{\eta \,\phi } \bigg ) }{{\sigma }^{2}\eta \,\ln \bigg ( \tau \bigg ) }}\\&\quad +2\,{\frac{C}{{\sigma }^{2}\eta \,\ln \bigg ( \tau \bigg ) \bigg ( -1+n{\tau }^{\eta \,\phi } \bigg ) }}-\frac{1}{2} \,{\frac{C\ln \bigg ( {\tau }^{\eta \,\phi } \bigg ) }{{\sigma }^{2}\eta \,\ln \bigg ( \tau \bigg ) }}+{\frac{C\phi }{\sigma }} \bigg ).\\ \end{aligned} \end{aligned}$$

Where $$\phi = \frac{\lambda x^\alpha }{\alpha }+(\frac{1}{\ln ( \tau )}\sqrt{-{\frac{2C}{G}}}) t$$.

## Discussion and graphs

This section digs into the soliton solutions uncovered during our investigation of the CSKMMS. We derive these soliton solutions using a different *r*+mEDAM approach, which provides us with a complete understanding of the CSKMMS’s complex dynamics. The diversity of soliton behaviour, notably shock and topological solitons, is efficiently represented through visual presentations.

Two significant types of solitons, shock and topological solitons, arise as unique magnetic field configurations and excitations in the field of ferromagnetism. Shock solitons are triangular and rectangular solitons that exhibit rapid magnetization shifts in various configurations as a result of external magnetic fields or magnetic domain interactions. These solitons emerge as a result of nonlinear magnetization dynamics guided by a balance of energy components including exchange interactions and anisotropy effects. Topological solitons, on the other hand, are stable, confined structures that originate from the need to accept changes in magnetization direction while reducing energy expenditures. Their topological properties provide their stability and distinctive behaviour, making them robust entities even in the absence of external disturbances inside ferromagnetic materials.

**Figure 1:**
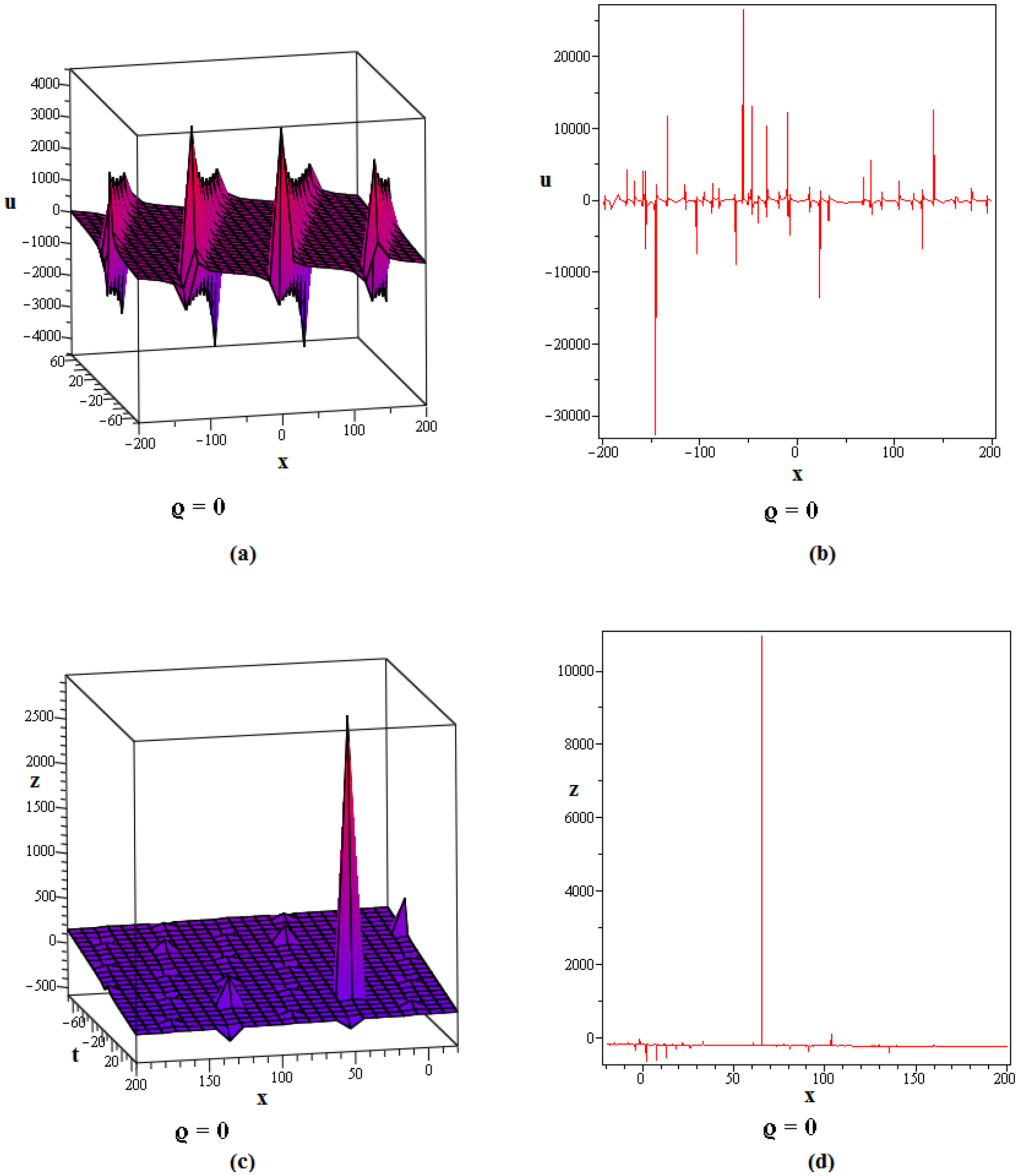
These figures of $u_{1,1}$ and $z_{1,1}$ in (21), are plotted with $j=8,k=5,l=2,r=50,\alpha =1,\tau ={\textrm{e}},\sigma =20, \lambda =2,C=-3$ respectively. Moreover, the 2D graphs are constructed simultaneously for $t=60$.

**Figure 2:**
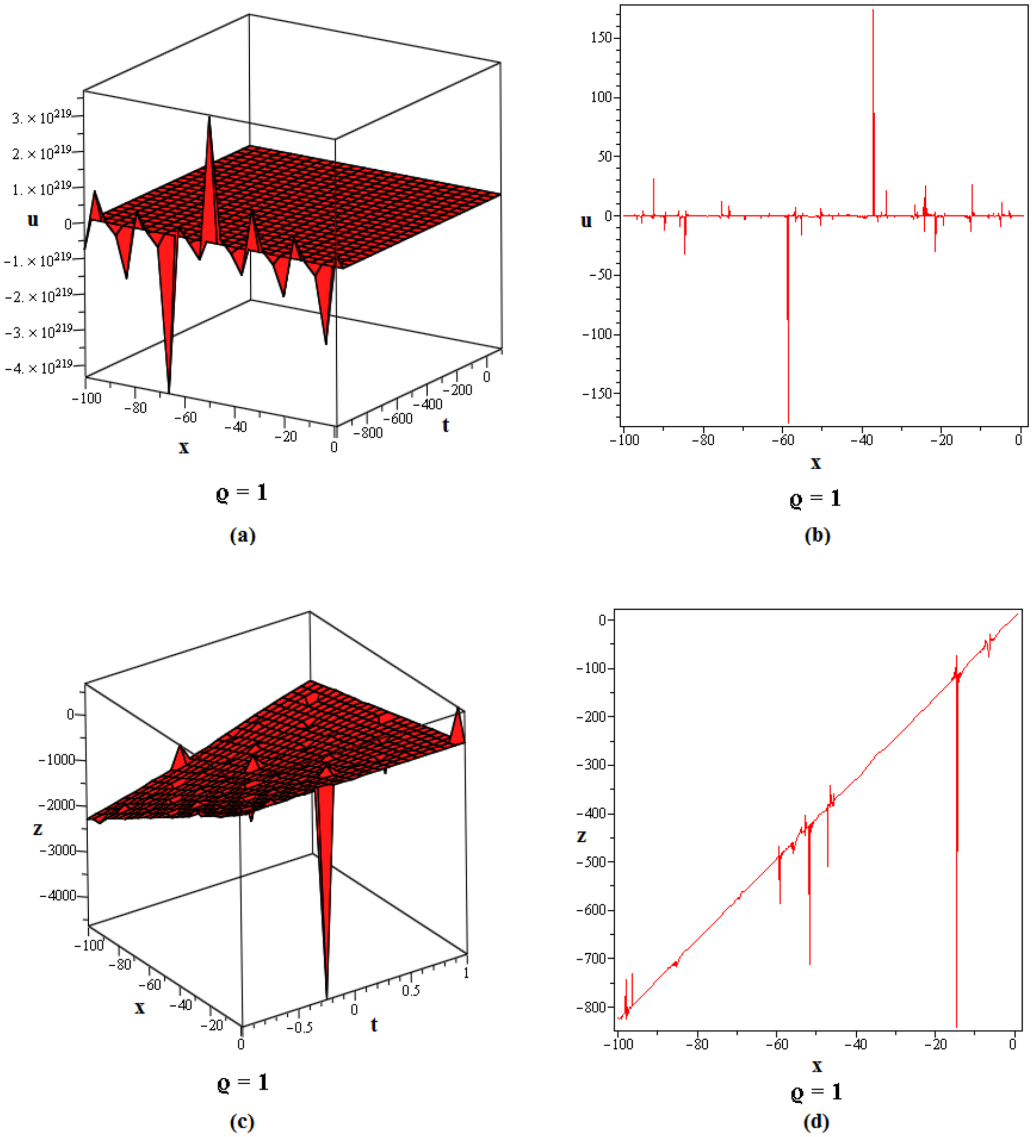
These figures of $u_{1,2}$ & $z_{1,2}$ in (22), are plotted with $j=2,k=2,l=4,r=10,\alpha =1,\tau ={\textrm{e}},\lambda =6,C=9,\sigma =5$ respectively. The 2D graphs are constructed simultaneously for $t=10$ and $t=1$ respectively.

**Figure 3:**
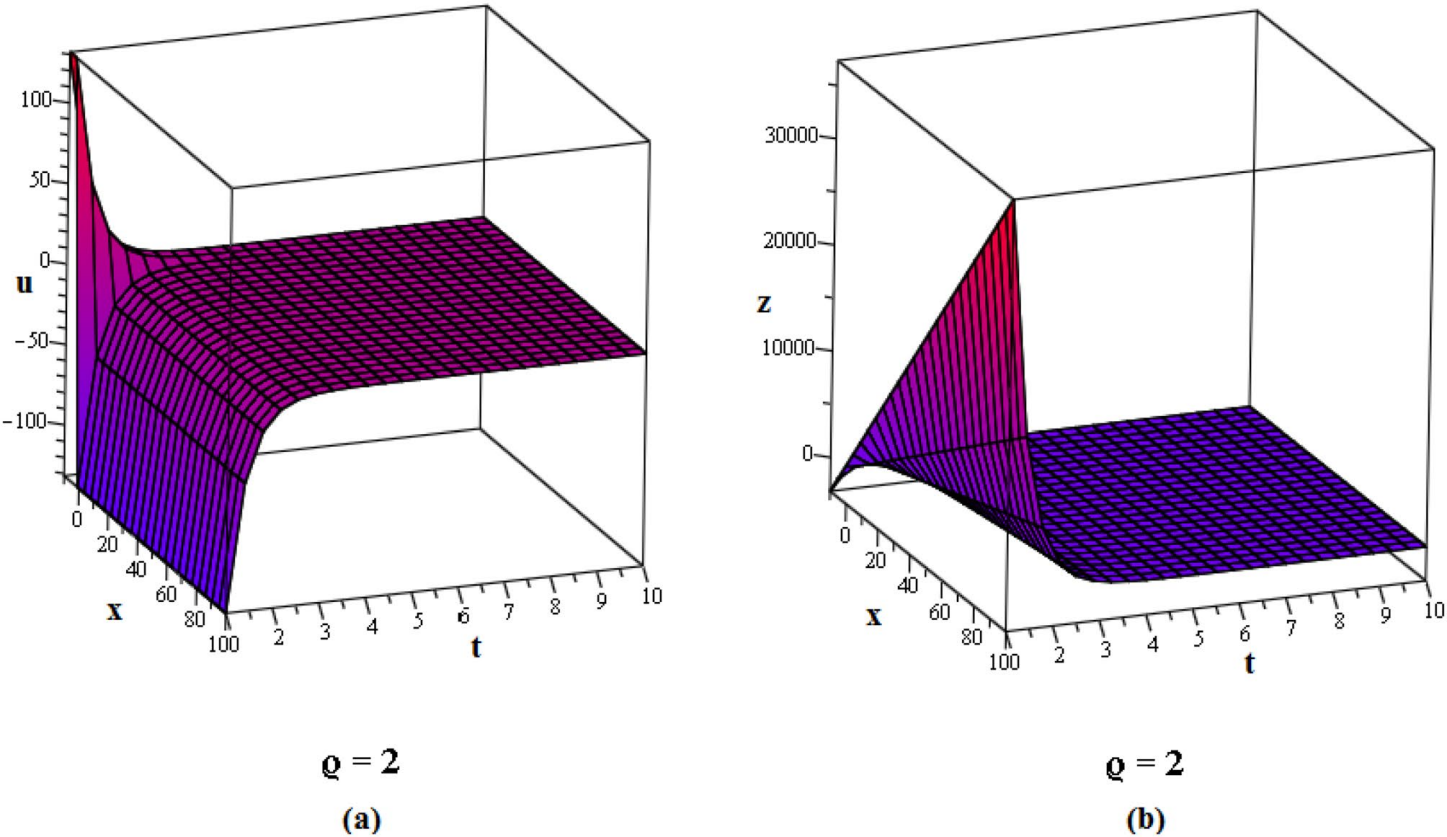
These figures of $u_{1,20}$ & $z_{1,20}$ in (40), are depicted with $j=8,k=0,l=-7,r=5,\alpha =1,\tau ={{ \mathrm e}},\lambda =30,C=-2, \sigma =40$ respectively.

**Figure 4:**
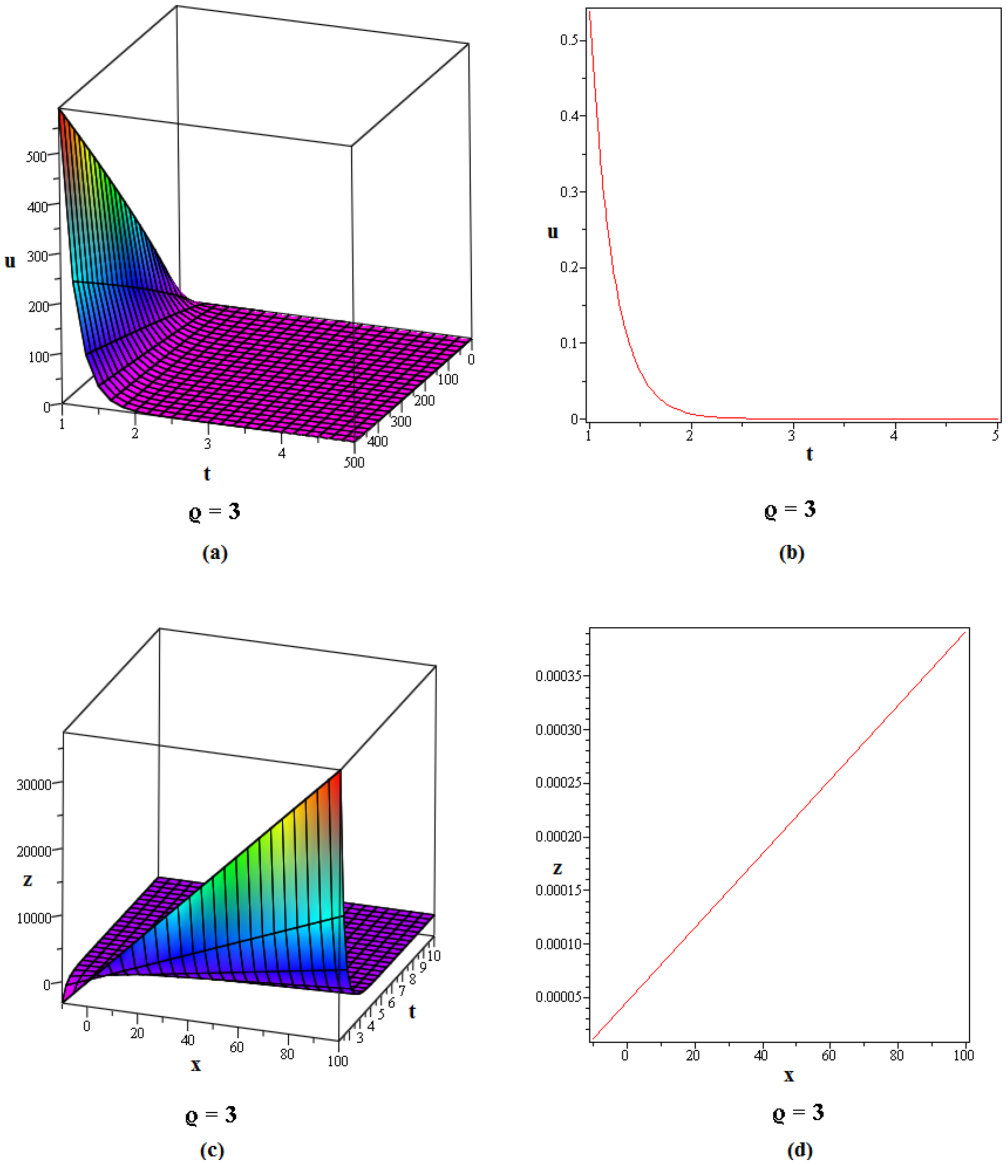
These figures of $u_{1,32}$ & $z_{1,32}$ in (52), are plotted with $j=6,k=2,n=3,\eta =2,l=0,r=100,\alpha = 0.9,\tau =2,\lambda =11,C=22,\sigma =3$. The 2D graphs are constructed simultaneously for $t=0$ and $t=10$ respectively.

**Figure 5:**
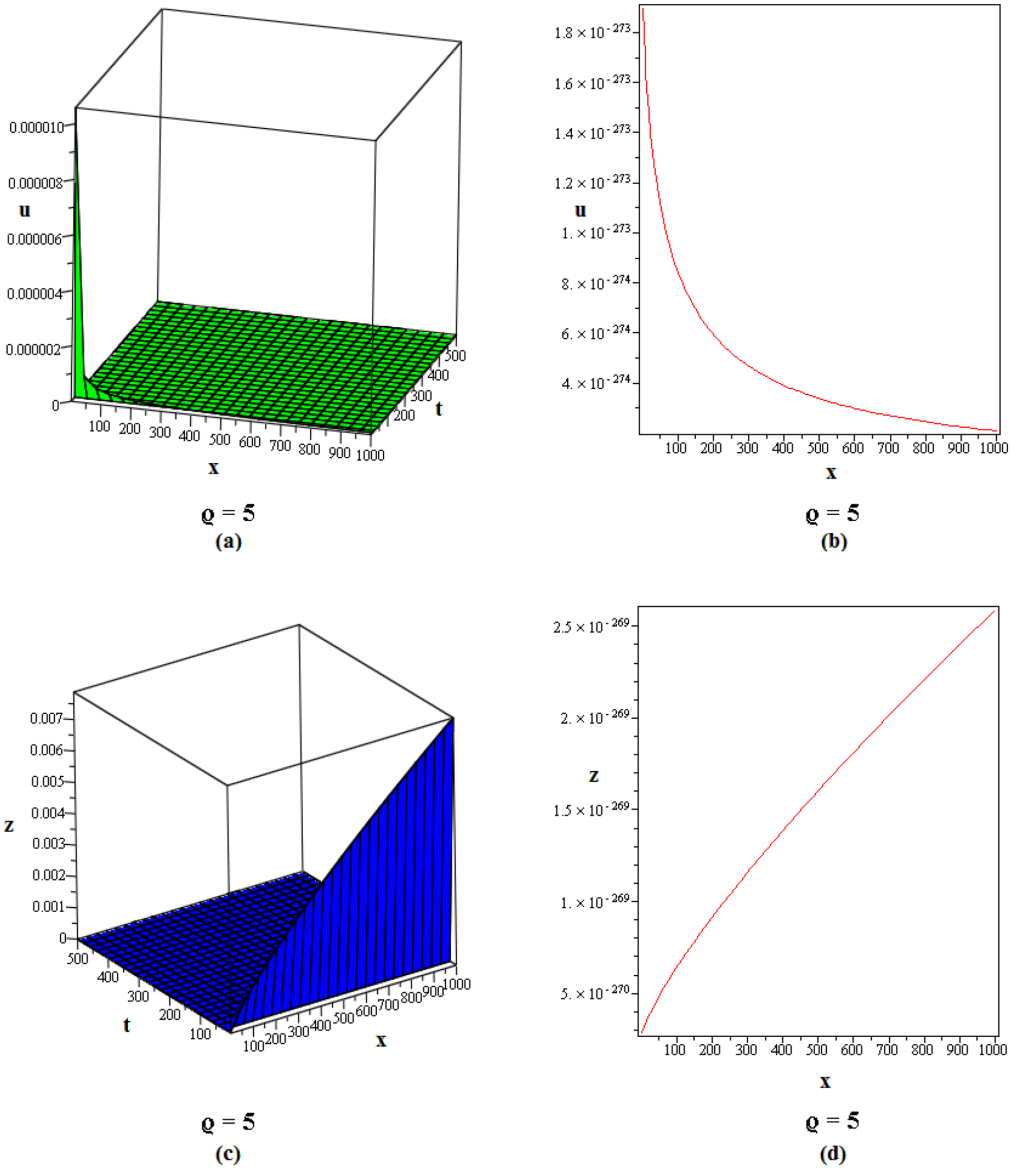
These figures of $u_{1,33}$ and $z_{1,33}$ in (53), are plotted with $j=0,k=0,l=20,r=-5,\alpha = 0.8,\tau =5,\sigma =-1,\lambda =-1,C=2$. The 2D graphs are constructed simultaneously for $t=50$ respectively.

**Figure 6:**
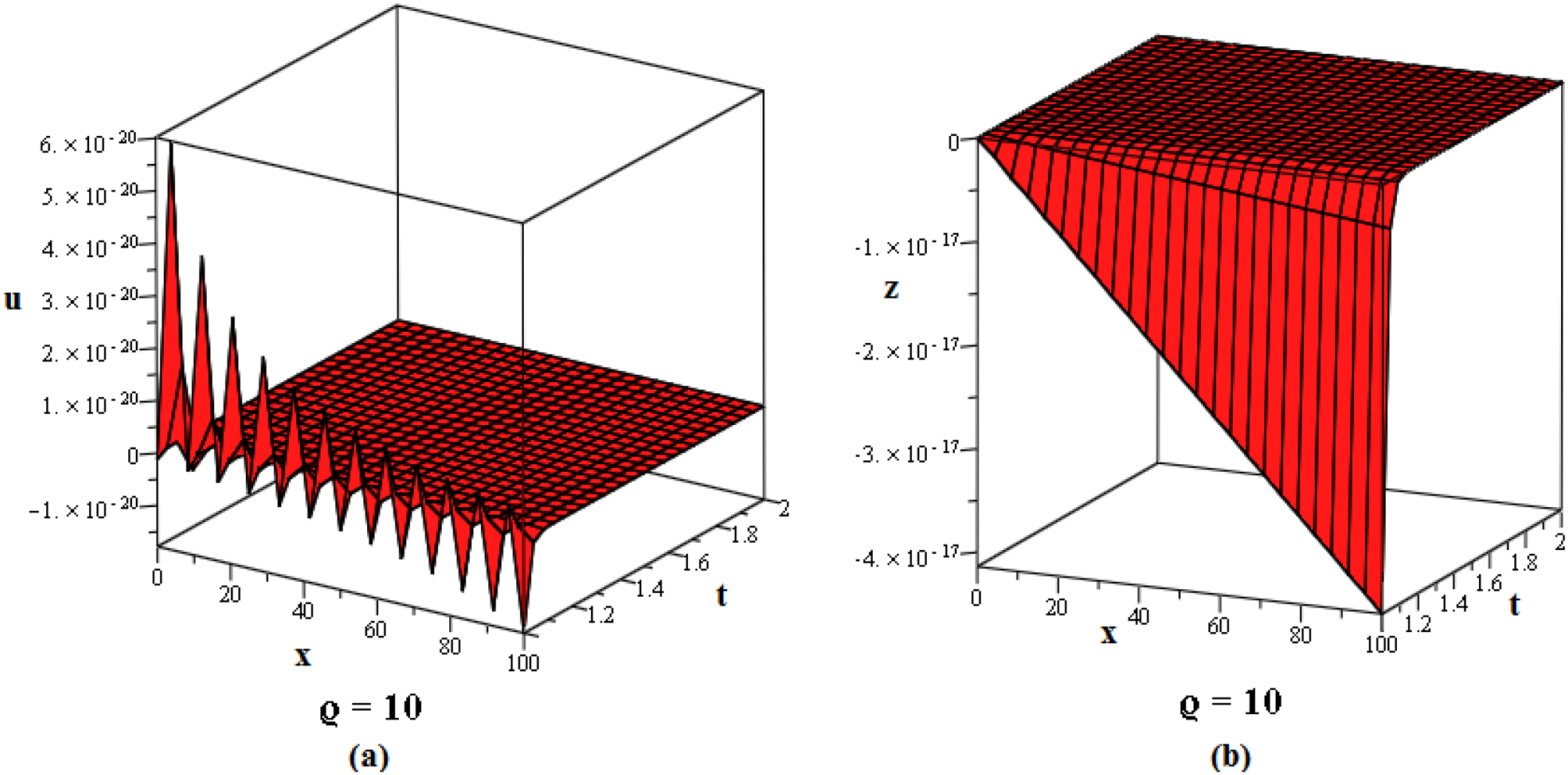
These figures of $u_{2,13}$ and $z_{2,13}$ in (69), are plotted with $j=5,k=0,l=30,r=45,\alpha =1,\tau ={\textrm{e}},\lambda =2,C=10$.

**Figure 7:**
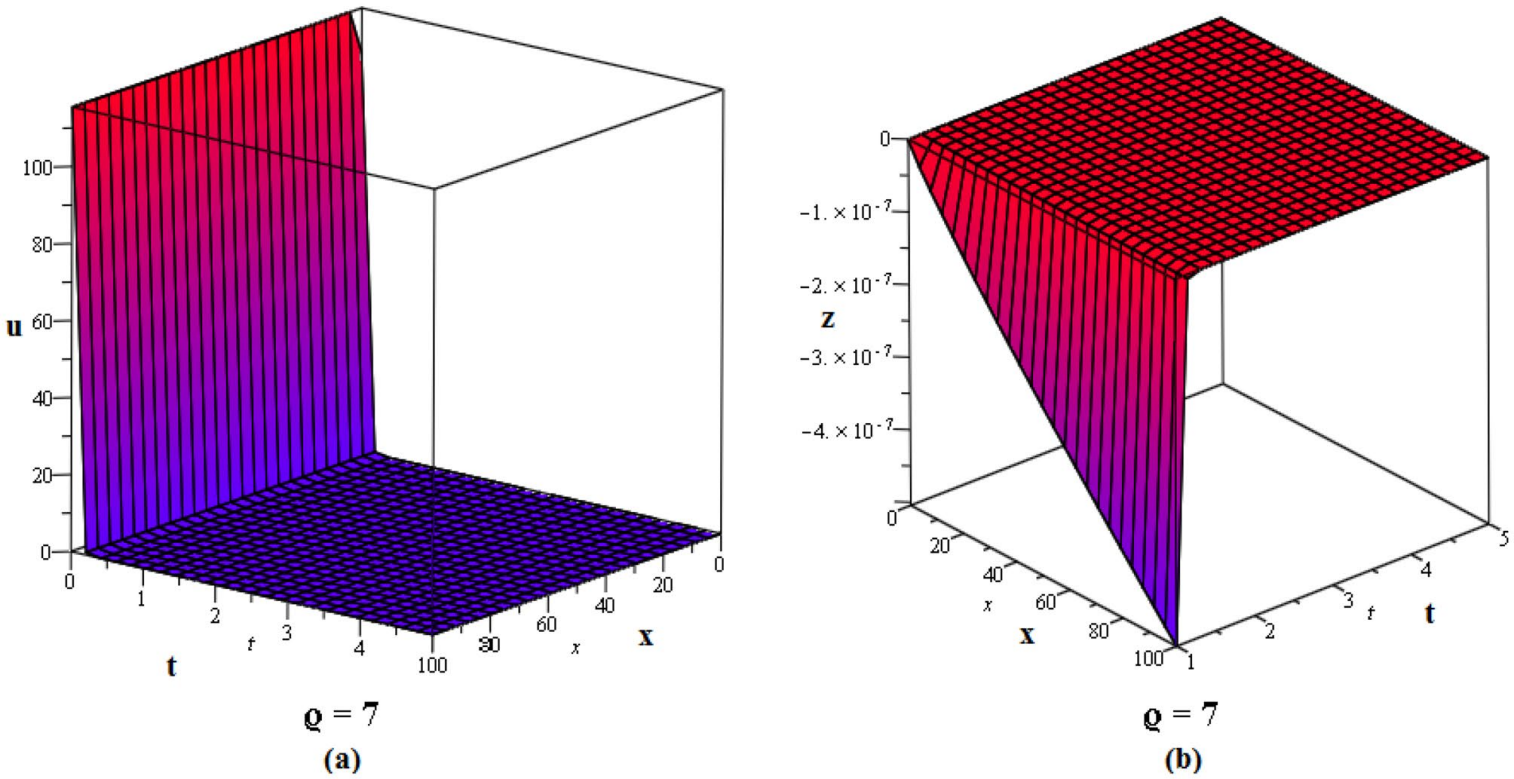
These figures of $u_{2,33}$ and $z_{2,33}$ in (90), are plotted with $j=0,k=3,l=3,n=3,\eta =3,r=4,\alpha = 0.9,\tau =5,\lambda =6,C=-9$.

Figure 1, these figures of $u_{1,1}$ and $z_{1,1}$ in (21), are plotted with $j=8,k=5,l=2,r=50,\alpha =1,\tau ={\textrm{e}},\sigma =20, \lambda =2,C=-3$ respectively. Moreover, the 2D graphs are constructed simultaneously for $t=60$. Figure 2, these figures of $u_{1,2}$ & $z_{1,2}$ in (22), are plotted with $j=2,k=2,l=4,r=10,\alpha =1,\tau ={\textrm{e}},\lambda =6,C=9,\sigma =5$ respectively. The 2D graphs are constructed simultaneously for $t=10$ and $t=1$ respectively. Figure 3, these figures of $u_{1,20}$ & $z_{1,20}$ in (40), are depicted with $j=8,k=0,l=-7,r=5,\alpha =1,\tau ={{ \mathrm e}},\lambda =30,C=-2, \sigma =40$ respectively. Figure 4, these figures of $u_{1,32}$ & $z_{1,32}$ in (52), are plotted with $j=6,k=2,n=3,\eta =2,l=0,r=100,\alpha = 0.9,\tau =2,\lambda =11,C=22,\sigma =3$. The 2D graphs are constructed simultaneously for $t=0$ and $t=10$ respectively. Figure 5, these figures of $u_{1,33}$ and $z_{1,33}$ in (53), are plotted with $j=0,k=0,l=20,r=-5,\alpha = 0.8,\tau =5,\sigma =-1,\lambda =-1,C=2$. The 2D graphs are constructed simultaneously for $t=50$ respectively. Figure 6, these figures of $u_{2,13}$ and $z_{2,13}$ in (69), are plotted with $j=5,k=0,l=30,r=45,\alpha =1,\tau ={\textrm{e}},\lambda =2,C=10$. Figure 7, these figures of $u_{2,33}$ and $z_{2,33}$ in (90), are plotted with $j=0,k=3,l=3,n=3,\eta =3,r=4,\alpha = 0.9,\tau =5,\lambda =6,C=-9$. To represent the interrelationships between different types of solitons, their propagation patterns, interactions, and the impact of noise on them, several 2D and 3D graphs are used. This visual analysis emphasises the relevance of our discoveries and verifies the *r*+mEDAM method’s efficiency in disentangling complex nonlinear systems. Furthermore, this visual depiction highlights the *r*+mEDAM approach’s significant contributions to unravelling complicated nonlinear events and improving our knowledge of solitonic behaviour in the field of CSKMMS. It is also clear that when the noise effect ($\varrho$) rises, topological solitons convert into shock waves. The shift from topological solitons to shock waves happens when the system’s noise effect increases, disturbing solitonic stability and forcing them to convert into shock wave patterns.

## Conclusion

Our research revolved around the (CSKMMS), a critical mathematical framework for comprehending ferromagnetic material phenomena. We have developed a systematic set of families of stochastic soliton solutions that include trigonometric, hyperbolic, and rational functions. These solutions can be used to define magnetic fields in zero-conductivity ferromagnets using the *r*+mEDAM.The effect of stochastic terms and noise on these soliton solutions has been extensively examined using Maple-generated 2D and 3D graphical representations. Our findings reveal complex relationships within the CSKMMS framework, indicating the impact of increasing noise levels. Notably, increased noise causes solitons to transform into shock waves, highlighting the sensitivity of soliton dynamics to noise and improving our understanding of magnetic field behaviour in ferromagnetic materials. In the future, we will delve deeper into the stochastic model, investigating additional dimensions and parameters to better understand its intricate dynamics. The method used in this study not only produces a large family of exact solutions, but it additionally delivers valuable insights into the model’s behaviour. As a result, avenues for further study and affluence of computational frameworks to more accurately represent the complexity that characterise ferromagnetic material phenomena are opened up.

## Data Availability

The data sets used and/or analysed during the current study available from the corresponding author on reasonable request.
